# Dear‐PSM: A deep learning‐based peptide search engine enables full database search for proteomics

**DOI:** 10.1002/SMMD.20240014

**Published:** 2024-08-27

**Authors:** Qingzu He, Xiang Li, Jinjin Zhong, Gen Yang, Jiahuai Han, Jianwei Shuai

**Affiliations:** ^1^ Department of Physics National Institute for Data Science in Health and Medicine Xiamen University Xiamen China; ^2^ Wenzhou Key Laboratory of Biophysics Wenzhou Institute University of Chinese Academy of Sciences Wenzhou Zhejiang China; ^3^ Oujiang Laboratory (Zhejiang Lab for Regenerative Medicine, Vision and Brain Health) Wenzhou Zhejiang China; ^4^ State Key Laboratory of Nuclear Physics and Technology School of Physics Peking University Beijing China; ^5^ State Key Laboratory of Cellular Stress Biology Innovation Center for Cell Signaling Network School of Life Sciences Xiamen University Xiamen Fujian China

**Keywords:** deep learning, inverted index, mass spectrometry, peptide search, proteomics

## Abstract

Peptide spectrum matching is the process of linking mass spectrometry data with peptide sequences. An experimental spectrum can match thousands of candidate peptides with variable modifications leading to an exponential increase in candidates. Completing the search within a limited time is a key challenge. Traditional searches expedite the process by restricting peptide mass errors and variable modifications, but this limits interpretive capability. To address this challenge, we propose Dear‐PSM, a peptide search engine that supports full database searching. Dear‐PSM does not restrict peptide mass errors, matching each spectrum to all peptides in the database and increasing the number of variable modifications per peptide from the conventional 3–20. Leveraging inverted index technology, Dear‐PSM creates a high‐performance index table of experimental spectra and utilizes deep learning algorithms for peptide validation. Through these techniques, Dear‐PSM achieves a speed breakthrough 7 times faster than mainstream search engines on a regular desktop computer, with a remarkable 240‐fold reduction in memory consumption. Benchmark test results demonstrate that Dear‐PSM, in full database search mode, can reproduce over 90% of the results obtained by mainstream search engines when handling complex mass spectrometry data collected from different species using various instruments. Furthermore, it uncovers a substantial number of new peptides and proteins. Dear‐PSM has been publicly released on the GitHub repository https://github.com/jianweishuai/Dear‐PSM.

## Graphical abstract

The full database search strategy proposed in this study expands the search scope to include all peptide sequences within the database, with peptide mass tolerances extending to several thousand Daltons. Dear‐PSM utilizes an inverted index algorithm to construct an index table for experimental spectra, enabling rapid searches, and employs deep learning algorithms for peptide validation. Moreover, Dear‐PSM supports up to 20 variable modifications per peptide sequence and considers all possible combinations of these modifications, significantly expanding the peptide search space.

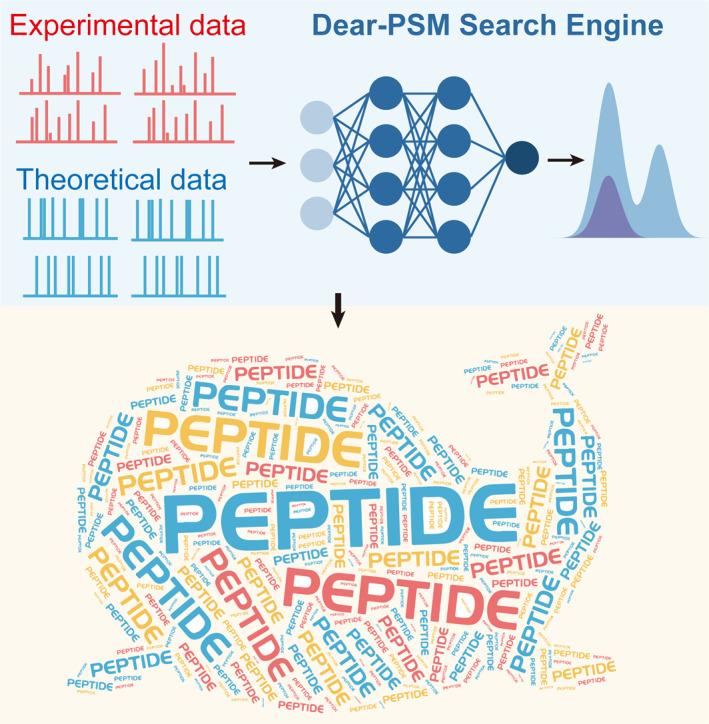

**Key points**

- Dear‐PSM leverages innovative techniques including inverted index technology and Deep Learning algorithms to achieve remarkable results
- Dear‐PSM increases the search range by 40‐fold, allowing peptide mass errors from −6000 to 4500 Da
- Dear‐PSM runs 3–7 times faster than mainstream search engines on regular desktops, with memory consumption reduced by 100–240 times

## 1 INTRODUCTION

Deciphering the amino acid sequence of peptides from extensive datasets and correlating them with respective proteins stands as a foundational pursuit in the realm of proteomics.^1^ Integral to this pursuit is the pivotal role played by mass spectrometry data analysis, notably through the widely adopted bottom‐up approach known as shotgun proteomics.^2^ This methodology involves enzymatic digestion of proteins into peptides and subsequent separation of resulting peptides via liquid chromatography‐tandem mass spectrometry (LC‐MS/MS), thereby generating mass spectrometry data reflective of peptide fragments.^3^, ^4^ Deep learning methods have also been widely applied in analyzing proteomic mass spectrometry data and other omics data in the field of bioinformatics.^5^, ^6^, ^7^, ^8^, ^9^, ^10^, ^11^

The prevalent approach in handling data‐dependent acquisition (DDA) proteomics data involves employing a search engine to sift through protein sequence databases. This search aims to align mass spectrometry data with potential peptide matches, determining the most likely candidates based on the search outcomes. Acting as an intermediary, the search engine operates by in silico digesting proteins and generates theoretical fragment spectra for peptides. It then compares these theoretical spectra with actual experimental data, evaluating the match and assigning a score to determine the peptide that best fits the experimental spectrum. This pivotal procedure, known as peptide‐spectrum matching Peptide spectrum matching (PSM), involves the intricate task of aligning peptide sequences with the captured mass spectrometry data.^12^

A single spectrum can match many potential peptides because different peptides may share the same fragments upon breaking apart. In shotgun proteomics, researchers commonly study post‐translational modifications (PTMs) in proteins, which occur after their creation. Incorporating PTMs in the database search significantly expands the pool of potential peptide candidates or matches between peptides and spectra. Standard search engines attempt to manage this complexity by setting boundaries on the allowed mass differences of peptides and the maximum number of modifications allowed in each peptide (typically three). However, these limitations can also hinder the search engine's ability to accurately interpret mass spectrometry data.

Search engines commonly used in shotgun proteomics fall into two main categories: narrow‐window search and open search strategies.^13^ The traditional narrow‐window ones, like Comet,^14^, ^15^ X! Tandem,^16^ MS‐GF+,^17^ Andromeda^18^ (part of MaxQuant^19^), MyriMatch,^20^ and OMSSA,^21^ set a range within which they hunt for candidate peptides based on the mass error tolerance for precursor ions. This range typically spans from 10 to 50 parts per million (ppm), tailored to the precision of various mass spectrometers.

However, a newer approach called open search has emerged, in which the mass error tolerance for precursor ions expands to hundreds of Daltons (Da), significantly broadening the scope for interpreting spectral data. This expansion has been instrumental in unveiling previously unidentified aspects of “dark matter” in shotgun proteomics. These advancements have led to the widespread adoption of open search across various proteomic applications. Some of the search engines supporting this approach include MSFragger,^22^, ^23^ Sage,^24^ pFind3,^25^ TagGraph,^26^ MetaMorpheus,^27^ and several others.

MSFragger stands out as the mainstream search engine in shotgun proteomics. It seamlessly integrates into the user‐friendly FragPipe workflow.^23^ By default, MSFragger operates within a mass error tolerance range of −150–500 Da for peptide precursor ions in open search mode. It also accommodates up to three variable modifications and allows for 5000 modification combinations per peptide.

However, as the number of variable modification increases, so does the exponential growth in candidate peptides and their corresponding fragment ions. This expansion makes constructing the MSFragger index time‐consuming and resource‐intensive, especially when considering modifications such as phosphorylation. In such cases, MSFragger's memory usage can skyrocket to hundreds of gigabytes, and the speed in open search mode notably decreases.

In this study, we propose a novel strategy called full‐database search. This strategy removes limitations on the mass tolerance of peptide precursor ions, broadening the search scope of each spectrum to encompass all peptides generated from the protein database through in silico digestion. We designed an experimental spectrum indexing algorithm to query the intersection of experimental spectra and theoretical spectra, and applied deep learning algorithms to score and verify the search results, which enabled the creation of a high‐performance peptide searching engine named Dear‐PSM. Unlike MSFragger, which necessitates a large theoretical fragment‐ion index, Dear‐PSM generates an experimental spectra index that usually requires only several hundred megabytes of memory, rendering it far more memory‐efficient than MSFragger. Within Dear‐PSM, we have implemented two matching scores, Hyper‐score and XCorrelation‐score (Xcorr score), and employed deep learning techniques to produce the final discriminated score. In contrast to MSFragger, which relies solely on hyper‐score, the dual‐score approach offers a more comprehensive means of identifying the best matching results. In addition, Dear‐PSM supports up to 20 variable modification sites per peptide, providing over a million potential modification combinations per peptide, covering most peptides with known modification sites. Dear‐PSM represents the first peptide search engine capable of full database searching, offering exceptional speed and minimal memory usage.

## 2 RESULTS

### 2.1 The principle of Dear‐PSM

Peptide spectral matching fundamentally involves the computation of intersections between experimental spectra and theoretical fragment ions derived from a peptide sequence. Subsequently, a scoring algorithm is applied to ions within this intersection to identify the most confidently matched results. In practice, peptide searches entail computing intersections between tens and hundreds of thousands of spectra and potential millions or even billions of theoretical peptide sequences.

MSFragger and Sage address this challenge by leveraging inverted indices of theoretical fragment ions, bypassing pairwise comparisons between peptides and experimental spectra, thereby significantly expediting the search process. Nevertheless, due to the large number of potential peptide sequences compared to experimental spectra, creating indices of theoretical fragment ions incurs considerable memory overhead, surpassing the capacity of regular computational platforms. To address this, we introduce a similar inverted index technology, establishing a fragment‐ion inverted index table tailored for DDA experimental spectra. This approach markedly reduces memory requirements while simultaneously enhancing search speed.

**FIGURE 1:**
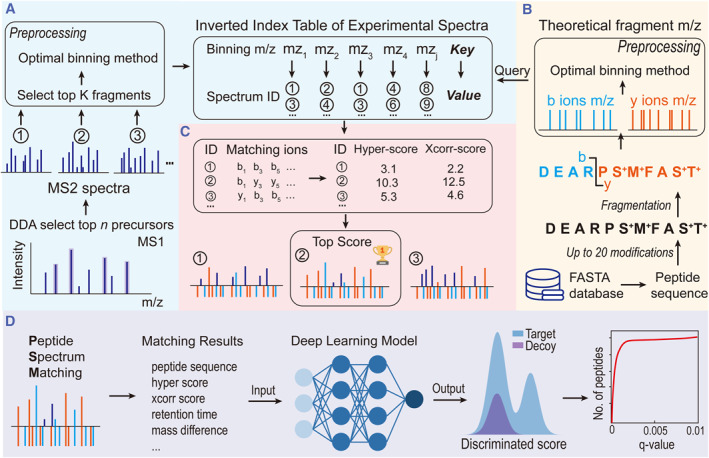
The principle of Dear‐PSM. (A) Creation of an inverted index for experimental spectra. Data‐dependent acquisition (DDA) experiments break the top n ranked MS1 precursor ions, generating MS2 spectra containing fragment m/z and intensities. Circled numbers denote unique identifiers (spectrum IDs) for MS2 spectra. The process of querying MS2 m/z to obtain spectrum IDs is termed inverted indexing. (B) Generation of theoretical fragment ions. Peptide sequences are enzymatically cleaved from the FASTA protein database and fragmented based on theoretical cleavage sites, producing all potential theoretical b and y ion m/z values. (C) Peptide spectrum matching process. Querying theoretical fragment m/z against the inverted index reveals matched b and y ions for each spectrum, and computing hyper‐score and xcorr‐score to generate the highest‐scoring match. (D) Peptide validation process. Employing a deep learning model to compute the final PSM score. The deep neural network uses information from the peptide spectrum matching results as input features, producing a discriminated score where higher scores indicate greater match confidence. Sorting by discriminant scores calculates *q*‐values for False Discovery Rate (FDR) filtering.

In conventional DDA experiments, the top‐n peptide ions (MS1) are fragmented to generate a series of fragment ion spectra (MS2). Dear‐PSM assigns a unique identifier to each MS2 spectrum, allowing retrieval of the corresponding fragment ion mass‐to‐charge ratios (m/z) via the spectrum ID. Subsequently, Dear‐PSM selects the K most intense fragment ions from the MS2 spectrum and discretizes the fragment ion m/z values into integers using an optimal binning algorithm (Supporting Information S1: Text S1). Then, Dear‐PSM constructs an inverted index table, enabling fast queries for experimental spectra containing specific fragment ions based on their binned m/z values (Figure 1A). Due to the significantly smaller number of experimental spectra compared to candidate peptides, the inverted index table typically requires only a few hundred megabytes of memory. The memory usage of the inverted index table is determined solely by the number of experimental spectra and is unaffected by the number of candidate peptide ions.

Next, Dear‐PSM traverses the protein database (FASTA database) to generate a series of peptide sequences based on theoretical enzymatic digestion. Subsequently, specific modifications set by the user are applied to these peptides, and theoretical b and y ions are generated in real‐time for these peptides during program execution (Supporting Information S1: Text S2). Finally, the optimal binning algorithm is employed to discretize the mass‐to‐charge ratios of each peptides b and y ions into integers, which serve as queries to the inverted index table to obtain the corresponding spectrum IDs (Figure 1B). Throughout this process, Dear‐PSM does not need to store all the b and y ions for each peptide, only retaining the matching results. This runtime generation of theoretical spectra significantly reduces memory usage.

The inverted index table expedites the retrieval of experimental spectrum IDs associated with each theoretical fragment‐ion, enabling matching peptide sequences with all spectra (Figure 1C). Importantly, this process obviates the necessity for precursor‐ion m/z values, enabling full‐database searches by computing intersections between individual peptides and all experimental spectra. Subsequently, leveraging the count and intensity of matched fragment ions, Dear‐PSM concurrently computes both hyper score and xcorr score as the PSM scoring metrics (Figure 1C). Obviously, each peptide matches numerous experimental spectra. For subsequent peptide validation, MSFragger retains and processes search results with over 4 matched fragment ions to compute the expected value, significantly increasing the computational time required. Dear‐PSM employs deep learning models for peptide validation, requiring solely the highest‐scoring PSM result and markedly reducing search time.

The output provided by Dear‐PSM encompasses peptide sequences, hyper‐scores, xcorr‐scores, retention times, and other pertinent feature data (Figure 1D). During the peptide validation phase, we first use 1% precursor‐level False Discovery Rate (FDR) threshold to filter out the target peptides as positive samples, and then we use all decoy peptides as negative samples to generate training samples for deep learning.^28^, ^29^ In proteomics, data collected from different mass spectrometers often follow different distributions. Therefore, we built a training set from each experimental dataset and trained the neural network from scratch to ensure consistent performance between the training and test sets. The training sets constructed from different experimental data and the trained neural networks are used exclusively within their respective datasets, ensuring no impact on other experimental data. The ultimate discriminant score was then computed via a deep neural network, serving as the conclusive metric for peptide‐spectrum matching (Figure 1D). Following the sorting of scores in descending order, Dear‐PSM outputs search results adhering to a 1% protein‐level FDR threshold (Figure 1D and Supporting Information S1: Text S3).

### 2.2 Benchmark testing datasets

To evaluate the performance of Dear‐PSM, we compared it with mainstream peptide search engines MSFragger and the latest open search engine, Sage. Performance benchmarking of Dear‐PSM was conducted using protein mass spectrometry datasets from various instruments and species, including the mixed‐species dataset^30^ PXD028735, the human species dataset^13^ PXD001468, and the human phosphorylation modification dataset^31^ PXD041271 (Supporting Information S1: Table S1). The protein sequence databases used in this study were obtained from UniProtKB/Swiss‐Prot and downloaded as per the required species.

Dear‐PSM conducts individual searches on DDA files, writing the search results into a single output file. Subsequently, deep learning was employed to score the search results, yielding filtered results at a 1% protein‐level FDR (Supporting Information S1: Table S2). MSFragger searches all DDA files and outputs search results individually. Then, Philosopher, included in FragPipe, validates peptides, and the search results are filtered using a 1% protein‐level FDR (Supporting Information S1: Table S3). Additionally, Sage's search results are similar to Dear‐PSM's, with all search results output into a single file. Sage utilizes its built‐in machine learning algorithm for peptide validation, resulting in filtered results at a 1% protein‐level FDR (Supporting Information S1: Table S4).

### 2.3 Deep learning models of Dear‐PSM

Dear‐PSM employs two deep learning models for predicting the peptide retention time and computing the discrimination score of PSMs. Since peptides exhibit relatively stable occurrence times, predicting peptide retention time can enhance peptide validation accuracy.^32^ During the retention time prediction process, Dear‐PSM first counts all the amino acids in the sequence, converting the peptide into a 20‐dimensional integer vector. Then, it counts the first two and last two amino acids of the sequence separately, generating two additional 20‐dimensional vectors. These three integer vectors are concatenated into a 60‐dimensional peptide vector. Additional features, such as the identity of the C‐terminal residue, peptide length, and mass, are appended, resulting in a 63‐dimensional feature vector that serves as the input for the deep neural network.

**FIGURE 2:**
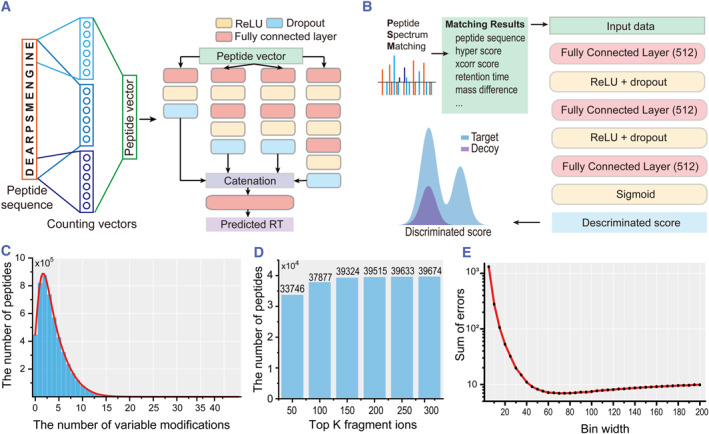
Deep learning model and key parameters of Dear‐PSM. (A) Deep learning model for predicting peptide retention time. It comprises a deep neural network with four branches, taking peptide sequences as input and converting them into peptide vectors by counting amino acids. The network output is the predicted retention time. The yellow, blue, and red boxes represent the ReLU activation function, Dropout layer, and Fully Connected layer, respectively. (B) Deep learning model for computing discriminant scores. (C) Distribution of the number of peptides in the human protein database with phosphorylation modification. (D) Impact of selecting fragment ion numbers on search results. (E) Sum of errors produced by different bin widths.

This method for converting peptide sequences into vectors can also be applied to mass spectrometry data with modifications. To enhance the predictive capability of the neural network, we referenced the Inception module in GoogleNet^33^ and designed a four‐branch neural network as the retention time prediction model (Figure 2A and Supporting Information S1: Figure S1). Subsequently, peptides filtered at 1% FDR were used as the training set to train the neural network. Furthermore, we optimized the model's structure, optimizer, and parameter quantity to achieve optimal performance (Supporting Information S1: Text S4).

The retention time predicted by the deep learning model is utilized as a feature for computing the PSM score. The PSM score is determined by various features reported in the search results. These features are combined into a vector and fed into a three‐layer fully connected network, which then produces the final PSM score (Figure 2B). To ensure accuracy, we calculated the threshold for 1% FDR by analyzing the distribution of discrimination scores between target and decoy peptides. Through comparative testing, we fine‐tuned the parameters of the fully connected network to achieve optimal performance (Supporting Information S1: Text S5).

### 2.4 The limitation of variable modifications

Protein modifications involve alterations in the mass of an amino acid within a protein, either an increase or decrease. During peptide searches, accounting for the mass changes due to modifications becomes crucial. Observations reveal an average amino acid count of 20 in theoretical peptides generated through in silico enzymatic cleavage. When exploring human samples for phosphorylation modifications, over 99% of peptides contained 20 or fewer variable modification sites (Figure 2C). For these peptides, Dear‐PSM supports up to 20 variable modifications, considering the complete combination of these modification sites. For peptides with *n* variable modifications less than 20, we consider 2^*n*^ possible modification combinations. For peptides with *n* greater than 20, we consider the number of possible modified peptides as *N* $=\sum_{i=0}^{3}C_{n}^{i}$​, where $C_{n}^{i}$ represents the number of combinations when randomly selecting *i* modifications from *n* sites. This decision was reached after conducting tests and balancing the number of candidate peptides with search time (Supporting Information S1: Text S2).

### 2.5 Data preprocessing of Dear‐PSM

During the data preprocessing step, Dear‐PSM first selects the top K ranked fragment ions in the experimental spectra, and then applies a data binning algorithm to convert the m/z of these K ions into integers for calculating the peptide‐spectrum intersections. Therefore, optimization is required for both the selection of the ion count K and the data binning algorithm. We chose a file from the PXD028735 dataset as our experimental test data.

Beyond 150 ions, the peptide count increased by only about 0.8% compared to selecting 150 ions (Figure 2D). Therefore, we set the default value for the selected ion count to 150. To validate the relationship between the error introduced by the binning algorithm and the actual instrument error, we computed the variance of errors for the test data at different bin widths (Supporting Information S1: Text S1). As the bin width increased, the variance initially decreased rapidly before slowly increasing, with the curve exhibiting only one minimum point (Figure 2E). This indicates the existence of a global optimal bin width that minimizes the binning error and closely approximates the actual instrument error.

### 2.6 Benchmark testing results

**FIGURE 3:**
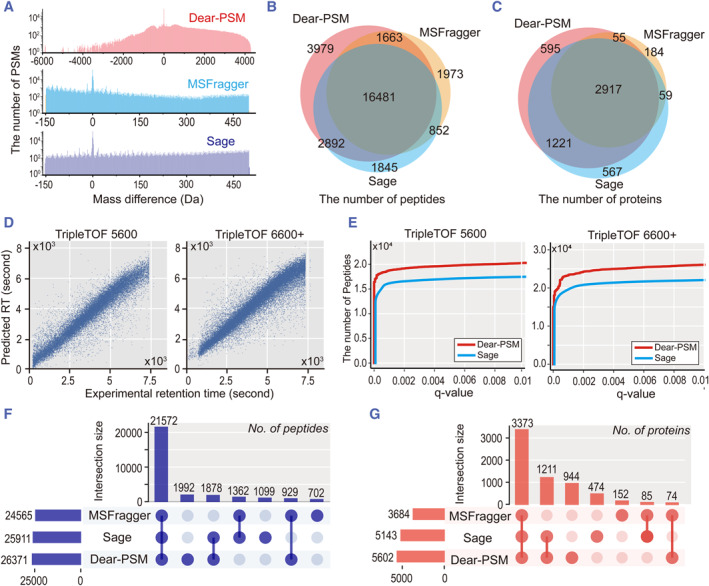
Benchmarked results of PXD028735 dataset. (A) Distribution of reported peptide mass differences by search engines. Red, blue, and purple denote Dear‐PSM, MSFragger, and Sage, respectively, based on data collected by Orbitrap QE HF‐X. Dear‐PSM utilizes the full‐database search strategy, while MSFragger and Sage employ open search strategies. (B) Peptides identified in TripleTOF 6600+ data under full‐database and open search strategies. (C) Proteins discovered in TripleTOF 6600+ data under full‐database and open search strategies. (D) Prediction of peptide retention time by Dear‐PSM using a deep learning model under full‐database search mode. (E) False discovery rate control curves. The red curve represents Dear‐PSM's false discovery rate curve under full‐database search mode, while the blue curve represents Sage's False Discovery Rate (FDR) curve under open search mode. (F) Upset^34^ plot of peptides discovered in TripleTOF 6600+ data under the narrow search strategy. (G) Upset plot of peptides discovered in TripleTOF 6600+ data under the narrow search strategy.

We first benchmarked the full‐database search mode using the Thermo Orbitrap data from the PXD028735 dataset. To compare the peptide search scopes of full‐database and open searches, we calculated the difference between the theoretical mass of peptides and their experimental mass, resulting in a distribution plot of mass differences. The MSFragger and Sage search engines were set to open search mode, with the default peptide mass search range from −150 to 500 Da. The search range of Dear‐PSM was extended from −6000 to 4500 Da, achieving comprehensive coverage of peptide sequences across the entire database. Sage reports PSM quality differences within the range of −150–500 Da, consistent with the default settings of open search (Figure 3A). We also examined Dear‐PSM's search scope, calculated Hyperscores, and Xcorr scores on data from other instruments (Supporting Information S1: Figure S2 and S3).

Next, we compared the numbers of peptides and proteins discovered through full‐database and open searches. For the data from TripleTOF 6600+, Dear‐PSM, MSFragger, and Sage reported 25,015, 20,789, and 22,070 peptides, respectively. Dear‐PSM discovered 20% more peptides than MSFragger and 13% more peptides than Sage (Figure 3B). Dear‐PSM, MSFragger, and Sage reported 4788, 3215, and 4764 proteins, respectively. The coverage of Dear‐PSM compared to MSFragger and Sage was 92% and 87%, respectively (Figure 3C). The full‐database search strategy of Dear‐PSM outperformed other search engines in the data collected from the TripleTOF 6600+ instrument. We also compared the number of peptides and proteins discovered by these three search engines on data from other instruments (Supporting Information S1: Figure S4 and S5).

Dear‐PSM improves validation accuracy by using a deep learning model to predict peptide retention times. Under the full‐database search mode, the predicted retention times exhibit a strong linear correlation with experimental times. Notably, for data from TripleTOF 5600 and TripleTOF 6600+, predicted values closely match the *y* = x curve, indicating the effectiveness of deep learning models in predicting peptide retention times (Figure 3D and Supporting Information S1: Figure S6). In addition, when examining the discriminant scores produced by Dear‐PSM on various instrument data, we observed a clear pattern: the scores assigned to target peptides formed two distinct peaks, while the scores for decoy peptides were predominantly clustered in the negative range. This clear separation suggests that the deep learning approach employed by Dear‐PSM is effective in distinguishing between genuine target peptides and decoys (Supporting Information S1: Figure S7).

Across different instrument data, Dear‐PSM's FDR curve trend mirrors Sage's, rapidly rising before stabilizing. This similarity suggests the reliability of Dear‐PSM's full‐database search strategy post‐peptide validation. Furthermore, Dear‐PSM discovers more peptides than Sage under various *Q*‐value conditions, indicating its ability to uncover a greater number of peptides (Figure 3E and Supporting Information S1: Figure S8). Dear‐PSM not only supports a full‐database search but also a conventional narrow window search. To further validate its peptide validation accuracy, we compared the peptide and protein counts discovered under the narrow window search mode among the three search engines. Specifically, for data from TripleTOF 6600+, Dear‐PSM, MSFragger, and Sage reported 26,371, 24,565, and 25,911 peptides, respectively (Figure 3F). The corresponding protein counts were 5602, 3684, and 5143. Dear‐PSM covered 94% and 89% of the results reported by MSFragger and Sage, respectively (Figure 3G). Dear‐PSM's peptide coverage exceeded 90% for both MSFragger and Sage, indicating its ability to replicate a substantial portion of the results from the other two search engines (Supporting Information S1: Figure S9).

**FIGURE 4:**
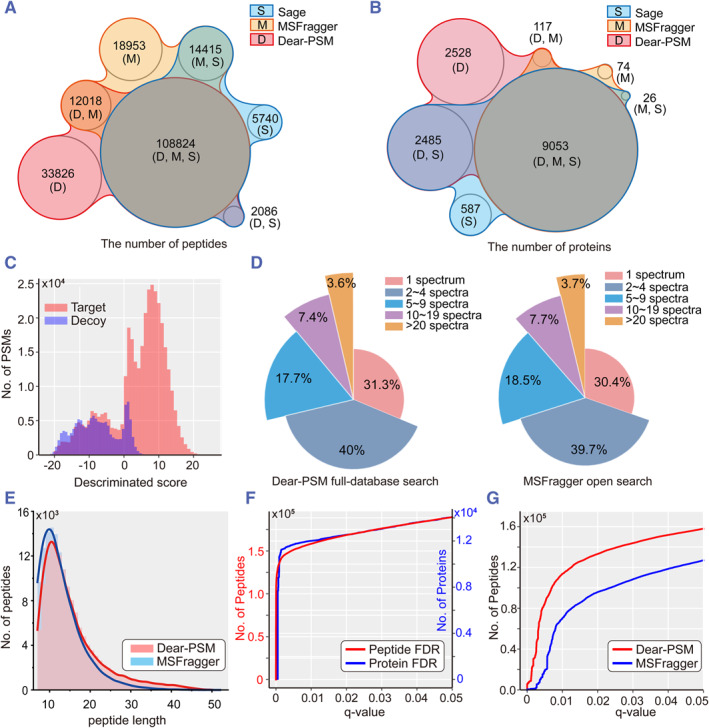
Benchmarked results of PXD001468 dataset. (A) nVenn^35^ plot shows the number of peptides found through full database search and open search strategies. (B) The number of proteins discovered through full database search and open search strategies. (C) Distribution of discriminant scores for PSMs output by Dear‐PSM using a deep learning model. Red and blue denote the discriminant scores for target and decoy peptides, respectively. (D) The proportion of spectra identified per peptide. The left subplot shows the results reported by Dear‐PSM in the full database search mode. The right subplot shows the results reported by MSFragger in the open search mode. (E) Histogram of peptide length distribution. The red and blue colors represent the distributions of Dear‐PSM and MSFragger, respectively. The curve represents the fitting distribution curve. (F) False Discovery Rate (FDR) control curve for Dear‐PSM. The red and blue curves represent the FDR curves for peptides and proteins, respectively. (G) Empirical FDR curve. The red and blue curves represent the number of identified peptides by Dear‐PSM and MSFragger respectively at different *q*‐values.

We benchmarked the full‐database search strategy using the larger dataset PXD001468. Under the open search strategy, Dear‐PSM, MSFragger, and Sage discovered 156,754, 154,210, and 131,065 peptides, respectively. Dear‐PSM achieved coverage rates of 78% and 85% compared to MSFragger and Sage, respectively. Additionally, Dear‐PSM uniquely identified 33,826 peptides, while MSFragger and Sage individually identified 18,953 and 5740 peptides, respectively (Figure 4A). Dear‐PSM, MSFragger, and Sage reported 14,183, 9270, and 12,151 proteins, respectively. Dear‐PSM achieved coverage rates of 99% and 95% compared to MSFragger and Sage, respectively. Furthermore, Dear‐PSM uniquely identified 2528 proteins (Figure 4B). The full‐database search strategy can uncover a significant number of peptides and proteins overlooked by traditional open searches.

We also evaluated Dear‐PSM's performance during the peptide validation phase under the full‐database search strategy. In the distribution plot of peptide discriminant scores, target peptides and decoy peptides are distinctly separated, with decoy peptide scores concentrated in the lower range (Figure 4C). Additionally, the predicted retention time also exhibited a linear correlation with the experimental retention time (Supporting Information S1: Figure S10). This illustrates the deep learning model's ability to differentiate between target and decoy peptides even with large datasets.

Typically, peptides identified by two or more spectra at the same time are more reliable. We checked how many spectra each peptide found by Dear‐PSM matched in the full database search mode. Among these peptides, 31.3% matched only one spectrum, 40% matched 2 to 4 spectra, and the rest matched 5 or more spectra (Figure 4D). This matches closely with what MSFragger reported, showing that Dear‐PSM's findings are trustworthy. We also looked at the length distribution of peptides reported by Dear‐PSM and MSFragger. Interestingly, Dear‐PSM found more peptides with over 20 amino acids compared to MSFragger (Figure 4E). This suggests that a full database search can uncover longer peptide sequences.

The peptide and protein FDR curves further demonstrate that the discriminant scores output by deep learning can effectively control FDR below 1% when dealing with large peptide volumes (Figure 4F). To further validate the FDR assessment accuracy of Dear‐PSM during peptide validation, we combined proteins from Arabidopsis thaliana and humans and searched the PXD001468 dataset using the merged FASTA database. We treated Arabidopsis proteins' corresponding peptides as decoys to calculate the Empirical FDR (Empirical FDR). Comparing Dear‐PSM's full‐database search mode with MSFragger's open search mode, we sorted Hyperscores in descending order to compute *Q*‐values. The FDR curves of Dear‐PSM and MSFragger exhibit consistent trends, with Dear‐PSM detecting more peptides than MSFragger at a 1% *q*‐value threshold under the full‐database search mode (Figure 4G).

**FIGURE 5:**
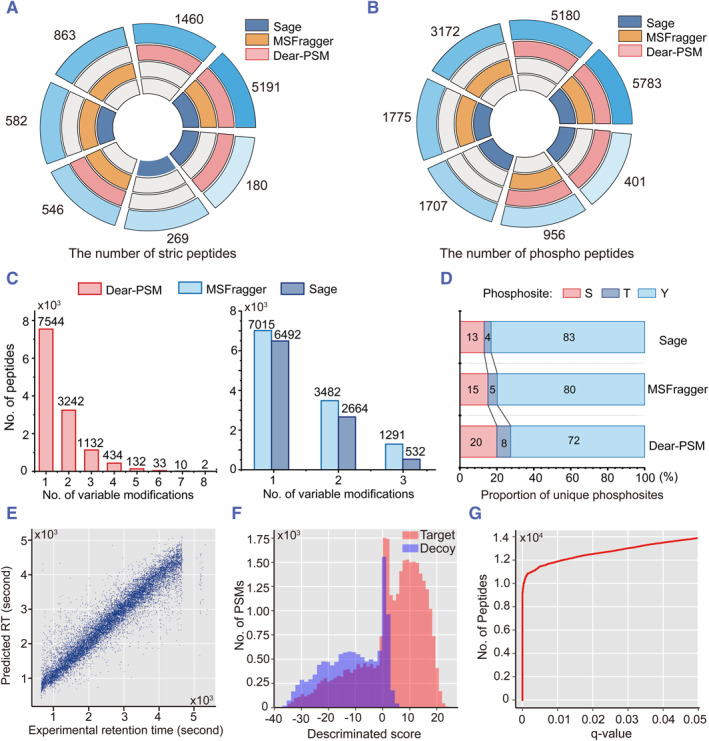
Benchmarked results of PXD041271 phosphorylated human dataset. (A) Results of strict peptide sequence comparison without modifications plotted by SuperExactTest.^36^ (B) Comparison results of phosphorylated peptides, where peptide sequences include phosphorylation modifications. (C) The number of variable modifications in peptides. Results from Dear‐PSM, MSFragger, and Sage are represented by red, light blue, and dark blue, respectively. (D) Proportion of phosphorylation sites. The proportion is calculated as the ratio of peptides containing only one type of site to the total number of phosphorylated peptides. Red, dark blue, and light blue boxes represent phosphorylation sites S, T, and Y, respectively. (E) Predicted retention times by deep learning. (F) Histogram showing the distribution of peptide discrimination scores output by deep learning. Red and blue bars denote target and decoy peptides, respectively. (G) Peptide false discovery rate control curve.

The full‐database search strategy expands the peptide mass error by 10‐fold, greatly increasing the pool of candidate peptides. Additionally, Dear‐PSM allows up to 20 variable modifications per peptide, resulting in a total of 2^20^ possible modification combinations. To assess the performance with the increased variable modification count, we conducted benchmark testing on the PXD041271 dataset, which includes phosphorylation modifications. Under the open search mode, Dear‐PSM, MSFragger, and Sage identified 7377, 7182, and 6222 peptides, respectively, when comparing unmodified peptide sequences (Figure 5A). Furthermore, Dear‐PSM covered 80% and 86% of the peptides discovered by MSFragger and Sage, respectively. When comparing phosphorylated peptides, Dear‐PSM detected 12,320 phosphorylated peptides, while MSFragger and Sage identified 11,686 and 9666 phosphorylated peptides, respectively (Figure 5B). Dear‐PSM reported 5% more phosphorylated peptides than MSFragger. However, Dear‐PSM also uniquely discovered 3246 phosphorylated peptides, attributable to the increased count of variable modifications.

Next, we compared the number of variable modifications in peptides reported by three search engines. Dear‐PSM, after increasing the upper limit of variable modifications, could identify peptides with up to 8 variable modifications in the PXD041271 dataset, whereas MSFragger and Sage could only detect peptides with a maximum of 3 variable modifications (Figure 5C). When comparing peptides with less than 3 variable modifications, the number of peptides reported by all three engines was similar. For instance, the number of peptides with 1 variable modification reported by Dear‐PSM, MSFragger, and Sage were 7544, 7015, and 6492, respectively (Figure 5C). Furthermore, we calculated the proportion of phosphorylation sites, defined as the ratio of peptides containing only one type of phosphorylation site to the total number of phosphorylated peptides. The proportion reported by Dear‐PSM was consistent with that reported by MSFragger and Sage, indicating that Dear‐PSM can be used to search for and validate phosphorylated peptides (Figure 5D).

Phosphorylation modification searches yielded peptide sequences differing significantly from standard datasets. Thus, we examined the peptide validation of Dear‐PSM on phosphorylation modification data. The retention time prediction remains linearly correlated with the experimental time, indicating its applicability to phosphorylation‐modified peptides (Figure 5E). The distribution of discrimination scores shows Dear‐PSM's ability to distinguish target and decoy peptides in the phosphorylation modification data (Figure 5F). Additionally, the FDR curve trend mirrors non‐phosphorylation data, demonstrating Dear‐PSM's suitability for FDR calculations in phosphorylation modification data (Figure 5G).

We tested the running performance of Dear‐PSM and MSFragger on a desktop computer. The test platform was equipped with an Intel Core i7‐7700K CPU (4 cores, 8 threads, 4.2 GHz), 64 GB of DDR4 2666 MHz memory, and a 2 TB solid‐state drive. We conducted tests using the PXD028735 dataset for Dear‐PSM's full database search and MSFragger's open search strategy. The results showed that Dear‐PSM identified 11,103,192 peptide sequences, whereas MSFragger identified 10,626,494 peptide sequences.

**FIGURE 6:**
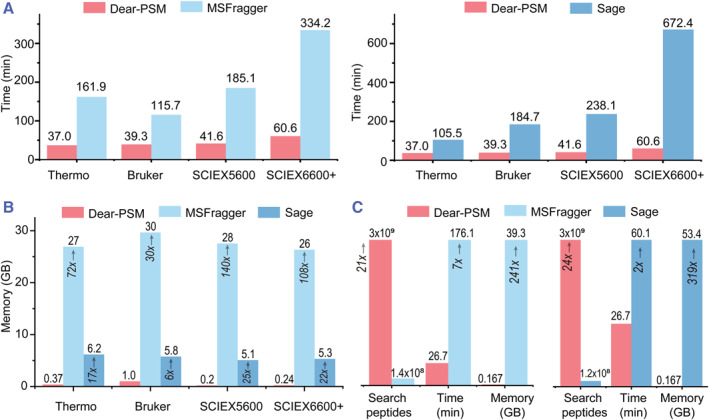
Benchmark test results of the running performance. (A) Comparison of search time on the PXD028735 dataset. (B) Comparison of memory usage during software runtime on PXD028735 dataset. (C) Comparison of search time and memory usage on PXD041271 dataset. Red, light blue and dark blue boxes in both (A), (B) and (C) represent Dear‐PSM MSFragger, and Sage, respectively.

Despite Dear‐PSM searching a peptide mass range 10 times larger than MSFragger, Dear‐PSM achieved a speed 3–6 times faster than MSFragger (Figure 6A). In terms of memory consumption, MSFragger used 30–136 times more memory than Dear‐PSM, while Dear‐PSM used an experimental spectral index table to greatly reduce memory consumption (Figure 6B). Additionally, Dear‐PSM searches are 3–11 times faster than Sage (Figure 6A). Although Sage saves approximately five times more memory compared to MSFragger, Dear‐PSM still consumes 6–25 times less memory than Sage (Figure 6B).

To further compare Dear‐PSM's performance in handling complex modification data, we tested it on the PXD041271 dataset containing phosphorylation modifications. After increasing the maximum number of variable modifications per peptide to 20, Dear‐PSM's search space expanded to 3 billion peptide sequences, while MSFragger's search space was 140 million peptide sequences (Figure 6C). Dear‐PSM's search space was approximately 21 times larger than that of MSFragger, reaching the order of 1 billion. Despite the immense search space, Dear‐PSM achieved a search speed 7 times faster than MSFragger and used 241 times less memory than MSFragger (Figure 6C). In addition, Dear‐PSM's search covers 24 times more candidate peptides than Sage, with a search speed that is twice as fast and memory savings of 319 times (Figure 6C). These results clearly indicate that Dear‐PSM outperforms existing mainstream search engines in terms of search space, speed, and memory efficiency.

## 3 CONCLUSIONS

This study introduces Dear‐PSM, a peptide search engine supporting full database searching for peptide identification in DDA data. Our full database search strategy extends the search scope to include all peptides in the FASTA database, with peptide mass errors expanded to several thousand Daltons. Dear‐PSM employs an inverted index algorithm for fast searching of experimental spectra and utilizes deep learning algorithms for peptide validation. Additionally, Dear‐PSM supports up to 20 variable modifications per peptide, significantly expanding the peptide search space.

Benchmarking results demonstrate that Dear‐PSM's full database search strategy enhances peptide identification and spectral interpretation capabilities. Dear‐PSM can reproduce results from MSFragger and Sage search engines in over 90% of cases in full database search mode, while also discovering more peptides and proteins. The use of deep learning for peptide validation in Dear‐PSM outperforms traditional machine learning algorithms and handles large datasets effectively. Moreover, deep learning algorithms can also handle phosphorylation modification data, expanding the applicability of Dear‐PSM. In performance comparisons, Dear‐PSM's search speed is 3–7 times faster than MSFragger, with memory consumption reduced by 100–200 times.

## 4 EXPERIMENTAL SECTION

Experimental details are provided in the Supporting Information.

## AUTHOR CONTRIBUTIONS

Conception and design, Qingzu He and Jianwei Shuai; algorithm development, software implementation and data analysis, Qingzu He; method discussion, Jianwei Shuai, Qingzu He, Xiang Li, Jinjin Zhong and Gen Yang; manuscript drafting, Qingzu He and Jianwei Shuai; study supervision, Jianwei Shuai and Jiahuai Han.

## CONFLICT OF INTEREST STATEMENT

The authors declare no competing interests.

## ETHICS STATEMENT

There are no experiments dealing with animal or human subjects or tissue samples from human subjects in this study.

## Supporting information

Supporting Information S1

## Data Availability

All comparative results from this research have been uploaded to the ProteomeXChange^37^ repository via the iProX^38^ partner repository with the iProX identifier. IPX0008601000. The login URL and password of the IPX0008601000 are “https://www.iprox.cn/page/SSV024.html;url=1714736539791Jgcv” and “Password: 2a6A”. To facilitate access and usage, the installation package for Dear‐PSM has been shared on the Github repository. Interested parties and researchers can conveniently access and download the software package from the following link: https://github.com/jianweishuai/Dear‐PSM.
