# Supplementary material for: Dear‐PSM: A deep learning‐based peptide search engine enables full database search for proteomics
Source: Smart Med. 2024 Aug 27;3(3):e20240014. doi: 10.1002/SMMD.20240014 (PMC11425048; doi:10.1002/SMMD.20240014)
Supplement: Supplementary file 1 — Supporting Information S1 [file SMMD-3-e20240014-s001.pdf]

Supplemental Information

Dear-PSM: a deep learning-based peptide search engine enables full database search for proteomics

Qingzu He, Xiang Li, Jinjin Zhong, Gen Yang, Jiahuai Han, and Jianwei Shuai

The PDF file includes:

Supplementary Figure S1 to S17

Supplementary Table S1 to S4

Supplementary Text S1 to S5

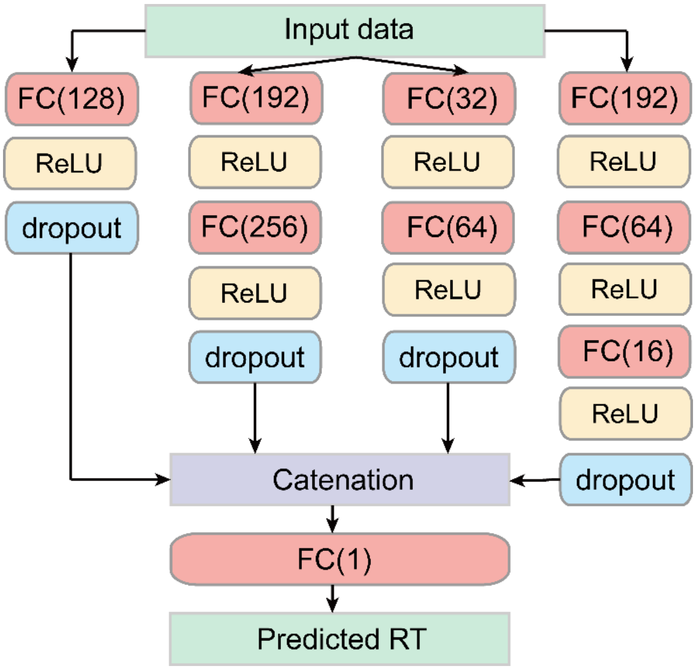

Figure S1. Deep learning model for predicting peptide retention time. The model consists of a four-branch network composed of fully connected layers (FC), with each branch accepting input data as a 60-dimensional vector. The numbers within the red boxes denote the dimensions of the fully connected layers. The yellow boxes represent ReLU activation functions, while the blue boxes denote dropout operations. The output vectors from the four branch networks are concatenated together through a concatenation operation. The final output of the network is a single-dimensional value representing the predicted retention time.

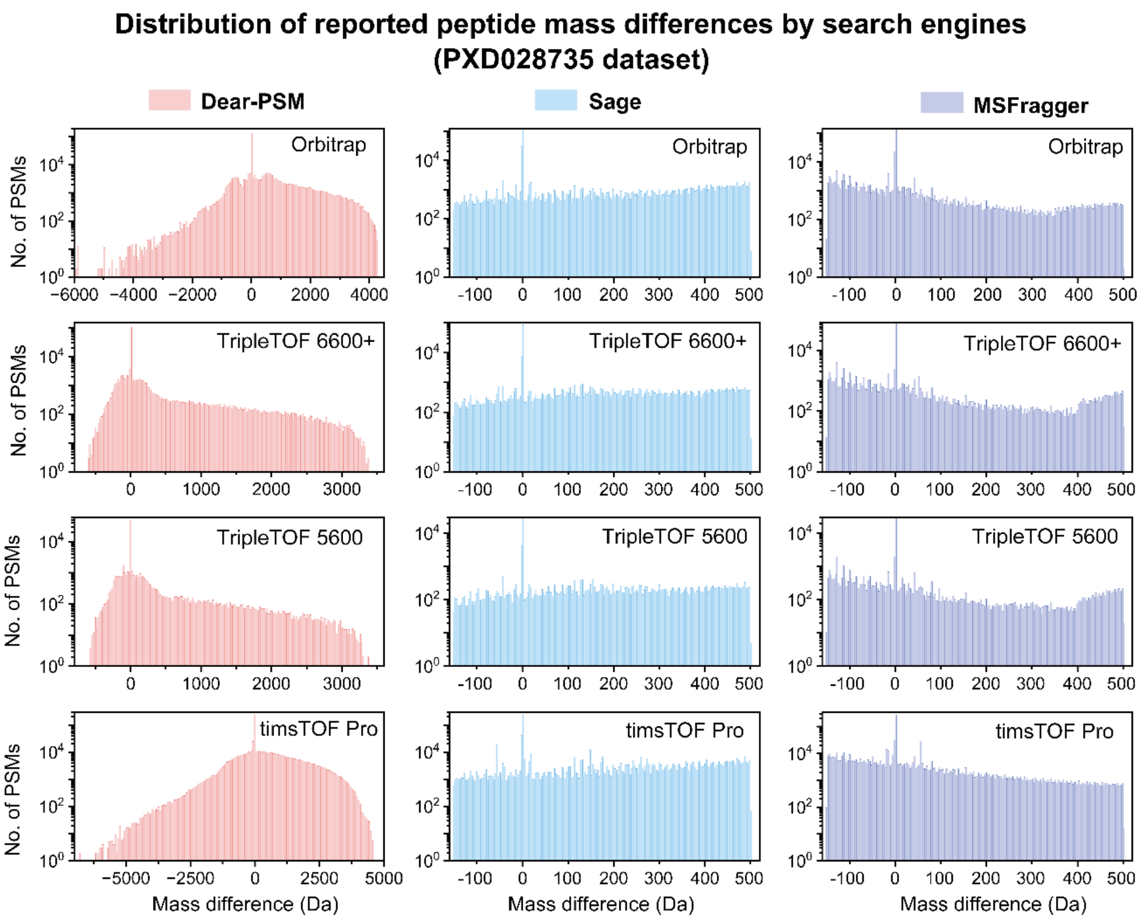

Figure S2. Distribution of peptide mass difference reported by search engines. The x-axis represents the mass difference, while the y-axis represents the number of peptide spectrum matches. The colors red, light blue, and purple represent Dear-PSM, Sage, and MSFragger respectively.

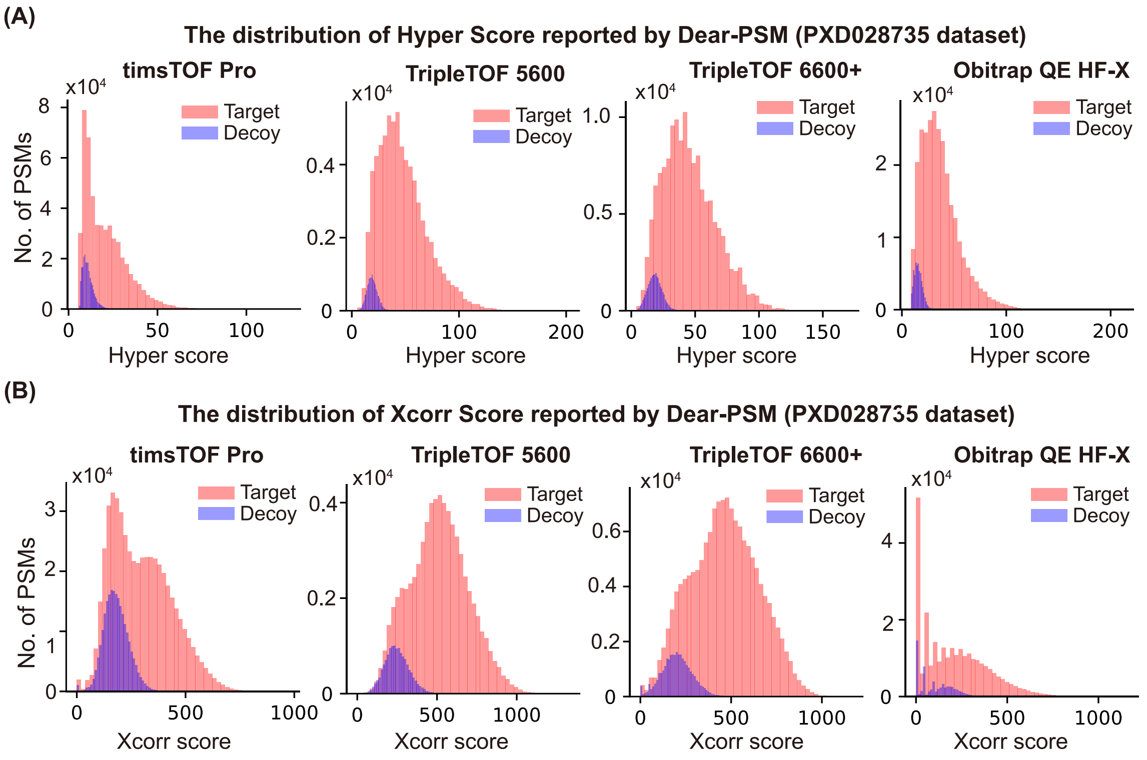

Figure S3. The Hyper score and Xcorr score outputed by Dear-PSM on full-database search mode. (A) Histogram showing the distribution of Hyper scores output by Dear-PSM. The horizontal axis represents the Hyper score, while the vertical axis represents the number of PSMs. (B) Histogram showing the distribution of Xcorr scores output by Dear-PSM. The horizontal axis represents the Xcorr score, while the vertical axis represents the number of PSMs. In both (A) and (B), red and blue colors respectively represent target peptides and decoy peptides.

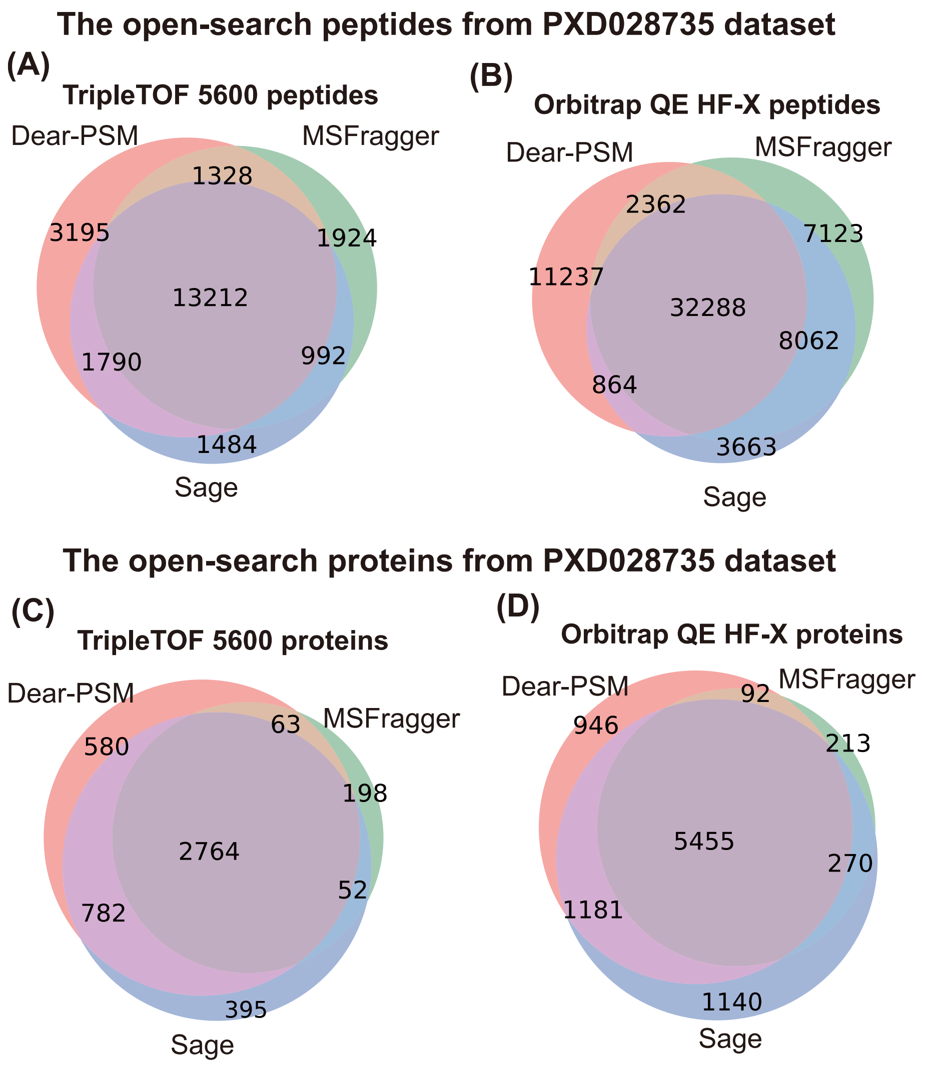

Figure S4. The number of peptides and proteins discovered by Dear-PSM on full-database search mode. (A) Peptides identified from TripleTOF 5600 data. (B) Peptides identified from Orbitrap QE HF-X data. (C) Proteins discovered from TripleTOF 5600 data. (D) Proteins discovered from Orbitrap QE HF-X data. The red, green, and blue circles represent the Dear-PSM, MSFragger, and Sage search engines respectively.

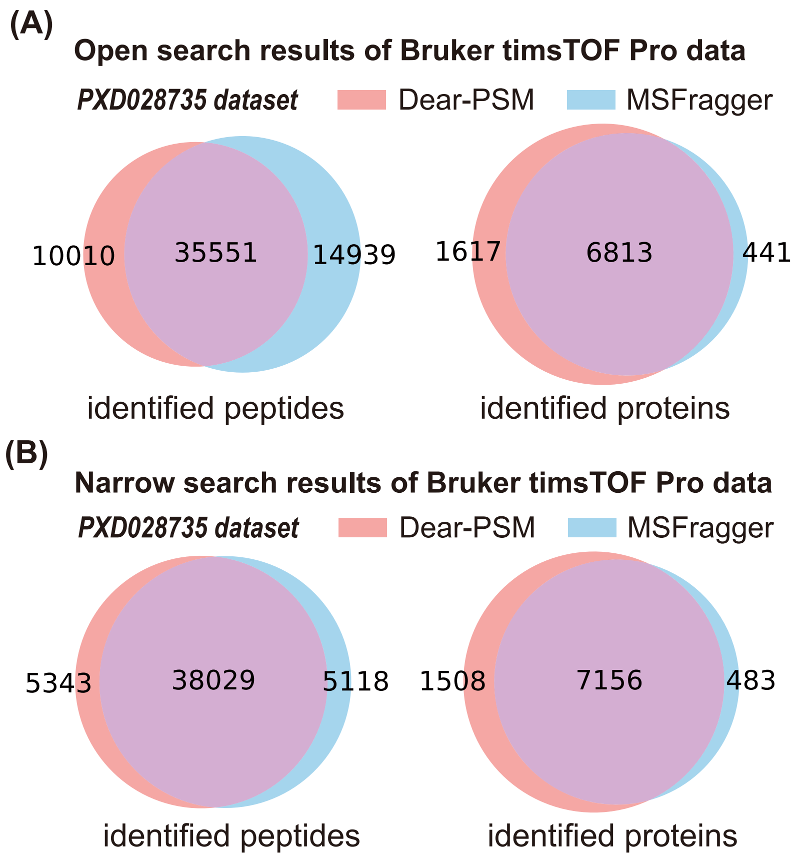

Figure S5. Comparison of results from Bruker timsTOF Pro data in open search and narrow window search modes. (A) Venn diagram showing peptides and proteins identified in open search mode, with red and blue representing Dear-PSM and MSFragger, respectively. (B) Venn diagram illustrating peptides and proteins identified in narrow window search mode, with red and blue denoting Dear-PSM and MSFragger, respectively.

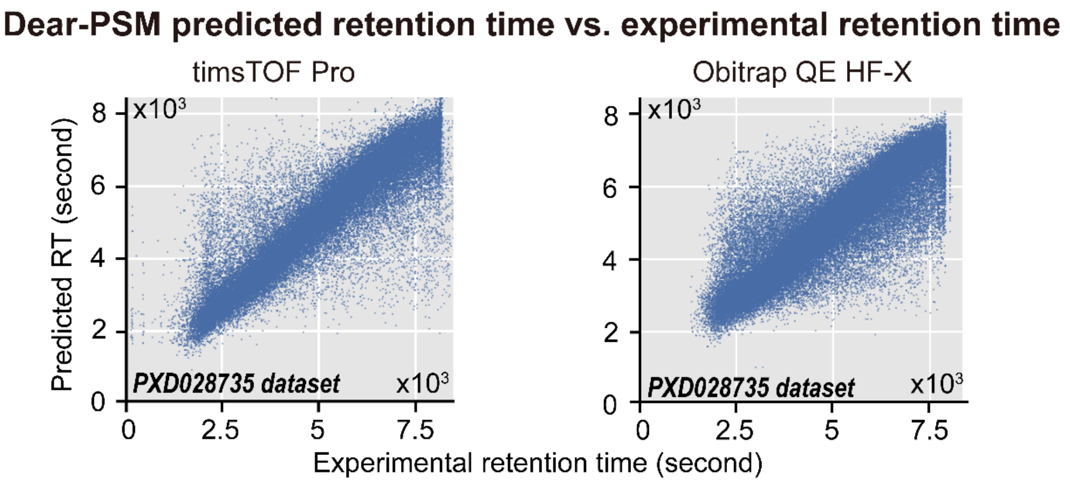

Figure S6. Correlation between predicted retention time by deep learning and experimental values. The horizontal axis represents the experimental retention time values, while the vertical axis represents the predicted retention time values by deep learning, both measured in seconds. The subplot titles denote four different experimental instruments.

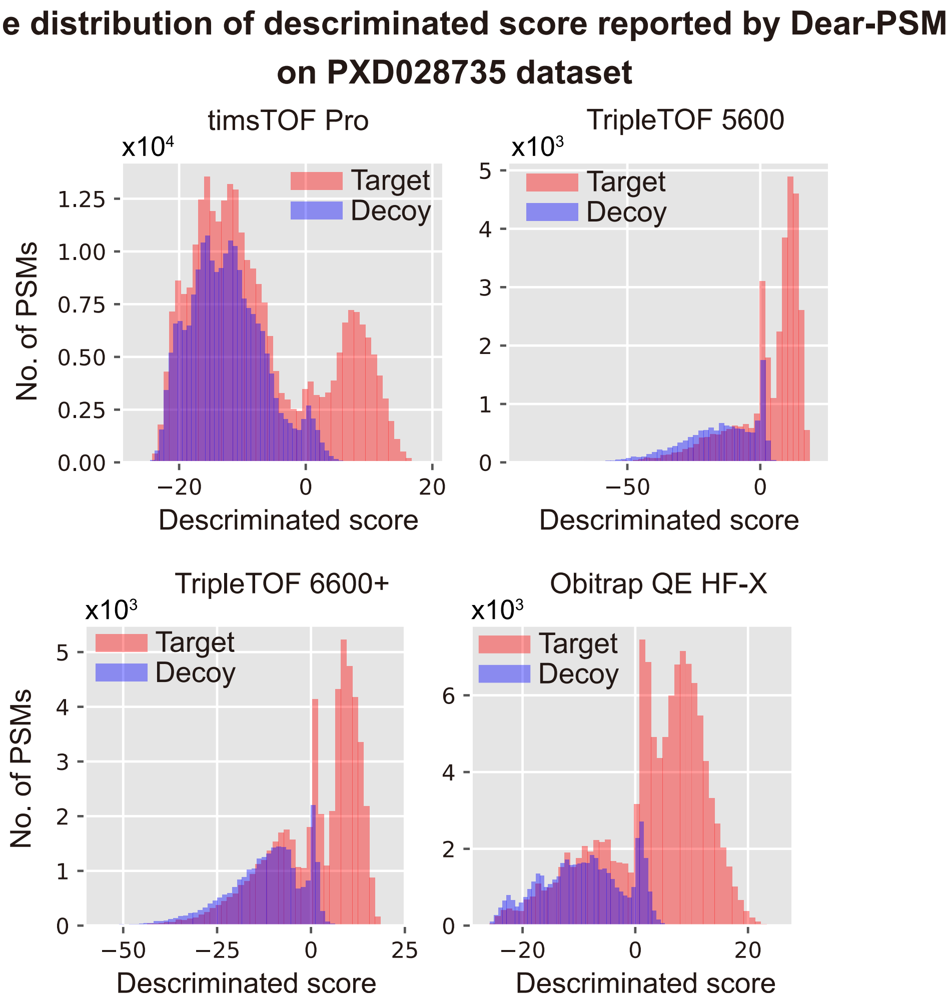

Figure S7. Histogram of peptide discrimination scores output by deep learning. The horizontal axis represents the discrimination scores outputted by deep learning, while the vertical axis represents the corresponding number of PSMs. Red and blue colors denote target and decoy peptides, respectively. The figure title indicates different experimental instruments.

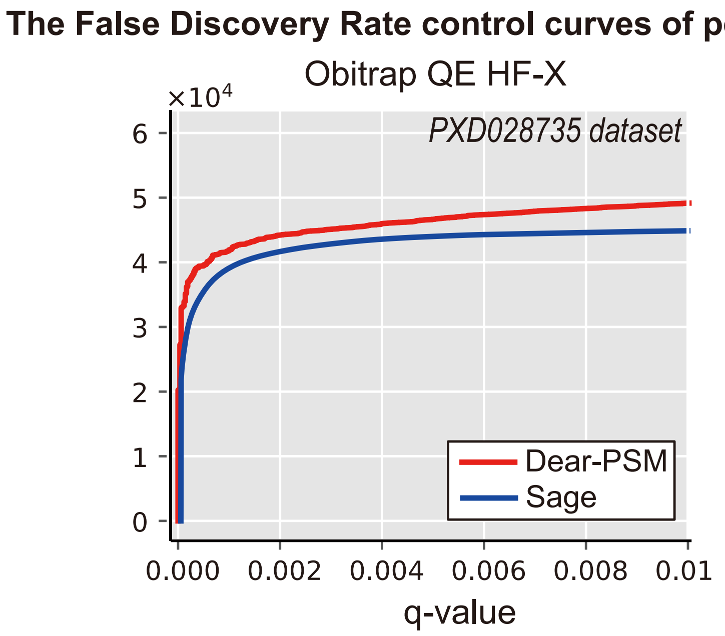

Figure S8. False discovery rate control curve of Orbitrap QE HF-X data. The red and blue curves represent the false discovery rate curves of Dear-PSM and Sage, respectively. The horizontal axis represents the q-value, and the vertical axis represents the number of peptides. The subplot titles indicate different models of experimental instruments.

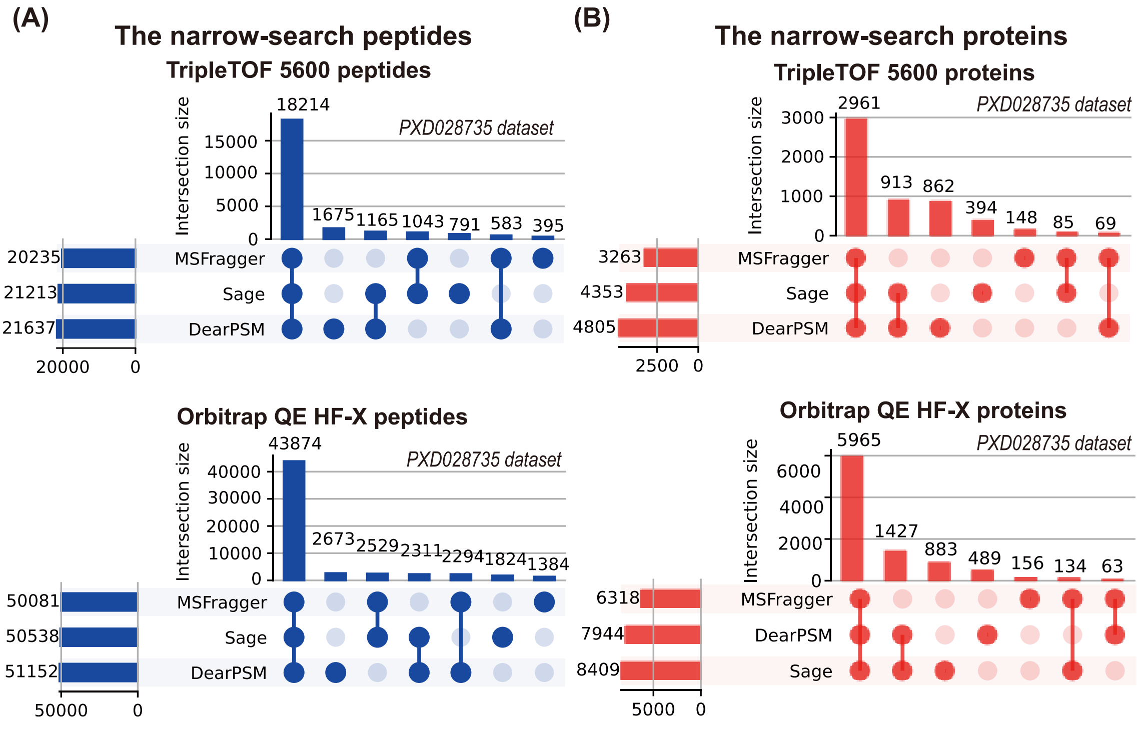

Figure S9. The number of peptides and proteins identified under narrow window search. (A) Peptides identified under narrow window search. (B) Proteins identified under narrow window search. Blue and red represent the search results for peptides and proteins, respectively.

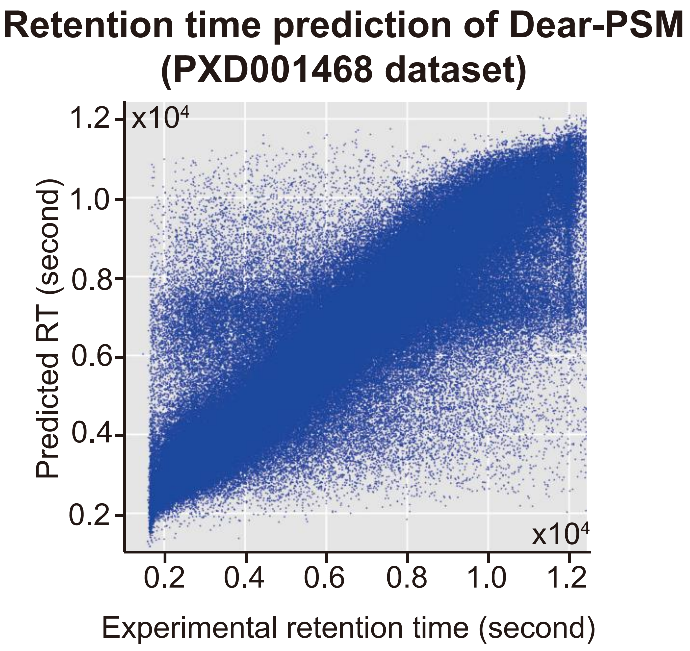

Figure S10. The correlation between experimentally recorded retention time values and retention time values predicted by deep learning. The horizontal axis represents the experimentally recorded retention time values, while the vertical axis represents the retention time values predicted by deep learning, both in seconds.

Table S1. Detailed Information of Benchmark Datasets.

Name	Species	Instrument	No. of Files	Total number of Spectra
PXD028735	Human, Yeast, *E. coli*	TripleTOF 5600<br>TripleTOF 6600+<br>Bruker TimsTOF Pro 2<br>Orbitrap QE HF-X	12	1,458,045
PXD001468	Human	Orbitrap Q Exactive	24	1,121,149
PXD041271	Human	Orbitrap Exploris 480	3	117,914

The benchmarking datasets used in this study were sourced exclusively from openly available datasets within the ProteomeXChange repository. Researchers can access the raw mass spectrometry data relevant to this study using the identification numbers PXD028735, PXD041274, and PXD013868.

To fairly compare software performance, we utilized the MSConvert tool to convert all test data into mzML files in centroid mode. Specifically, for mass spectrometry data acquired from ThermoFisher and Bruker TimsTOF instruments in .raw and .d formats, we employed the “vendor” algorithm within MSConvert’s “Peak Picking” option to transform them into mzML files. As for data obtained from SCIEX 5600 and SCIEX 6600 instruments in wiff format, we used qtofpeakpicker.exe for conversion to mzML files.

The PXD028735 dataset serves as a comprehensive benchmark for modern proteomics data acquisition strategies, particularly Label-Free Quantification (LFQ), offering quality control for bioinformatics algorithm development and evaluation. We selected QC data from this dataset, acquired using the DDA strategy, featuring a mixture of 65% human proteins, 22.5% yeast proteins, and 12.5% Escherichia coli proteins. These QC data were collected on four different instruments: SCIEX TripleTOF 5600, TripleTOF 6600+, Thermo Orbitrap QE HF-X, and Bruker timsTOF Pro, with three files chosen from

each instrument for benchmark testing, totaling 12 files for analysis.

The PXD001468 dataset originates from human HEK293 cells and was analyzed using the Orbitrap Q Exactive mass spectrometer. In each data acquisition cycle, the Orbitrap conducted a full MS scan (300-1500 m/z) and selected the 20 most abundant ions for isolation and fragmentation.

The PXD041271 dataset involves the enrichment of phosphorylation by treating HeLa cells stimulated with vanadate and serum using magnetic bead processors and phospho-tyrosine binding agents or antibodies, achieving a phosphorylated peptide enrichment efficiency exceeding 99%. Samples were analyzed using the Orbitrap Exploris 480 mass spectrometer.

Table S2. Parameter settings of Dear-PSM on different datasets.

Parameter Name	TTOF5600	TTOF6600	Orbitrap	timsTOF	PXD001468	PXD041271
ms1_tol_ppm (narrow search)	50	50	10	15	10	10
ms1_tol_ppm (full search)	-1	-1	-1	-1	-1	-1
ms2_tol_ppm	100	100	20	50	20	20
ms1_max_charge	4	4	4	4	4	4
modifications	42.01@n 57.02@C 15.99@M	42.01@n 57.02@C 15.99@M	42.01@n 57.02@C 15.99@M	42.01@n 57.02@C 15.99@M	42.01@n 57.02@C 15.99@M	42.01@n 57.02@C 15.99@M 79.97@STY
cut_after/skip	KR/P	KR/P	KR/P	KR/P	KR/P	KR/P

"ms1_tol_ppm" indicates the ppm-based mass error of peptide parent ions. This parameter is exclusively applicable in the narrow window search mode, while in the full database search mode, it is set to -1 to signify the absence of parent ion mass error.

"ms2_tol_ppm" denotes the ppm-based error in m/z for fragment ions.

"ms1_max_charge" signifies the maximum charge of peptide parent ions. If the DDA data does not furnish parent ion charge, this value is automatically adopted; otherwise, the charge value from the DDA data is utilized.

"modifications" denotes the modification settings, formatted as "mass@position". For example, "57.02@C" indicates a fixed modification at position C. Common variable modifications include "42.01@n" and "15.99@M", while the search for phosphorylation modifications in the PXD041271 dataset necessitates the addition of "79.97@STY".

"cut_after/skip" represents the theoretical enzyme cleavage site and the site to skip cleavage, with the default cleavage site being "KR" and the skip site being "P".

Table S3. Parameter settings of MSFragger on different datasets.

Parameter Name	TTOF5600	TTOF6600	Orbitrap	timsTOF	PXD001468	PXD041271
precursor mass tolerance (narrow search)	-50~50	-50~50	-10~10	-15~15	-10~10	-10~10
precursor mass tolerance (open search)	-150 Da~ 150 Da	-150 Da~ 150 Da	-150 Da~ 150 Da	-150 Da~ 150 Da	-150 Da~ 150 Da	-150 Da~ 150 Da
fragment mass tolerance	100	100	20	50	20	20
modifications	42.01@n 57.02@C 15.99@M	42.01@n 57.02@C 15.99@M	42.01@n 57.02@C 15.99@M	42.01@n 57.02@C 15.99@M	42.01@n 57.02@C 15.99@M	42.01@n 57.02@C 15.99@M 79.97@STY
Cleavage/Enzyme	trypsin	trypsin	trypsin	trypsin	trypsin	trypsin

"Precursor mass tolerance" denotes the mass error of peptide parent ions, measured in ppm. In the open search mode, it is set to the default range of -150 Da to 500 Da.

"Fragment mass tolerance" represents the mass error of fragment ions, also measured in ppm.

"Modifications" indicates the modification settings, where commonly used variable modifications include "42.01@n" and "15.99@M". When searching for phosphorylation modifications in the PXD041271 dataset, the modification position "79.97@STY" needs to be added.

"Cleavage/Enzyme" denotes the theoretical enzyme cleavage settings for peptides, set to the default trypsin configuration.

We used FragPipe workflow to validate the MSFragger search results. Within FragPipe, parameters for peptideprophet in open search mode include "--nonparam --expectscore --decoyprobs --masswidth 1000.0 --clevel -2". Conversely, parameters for

the narrow window search mode are “--decoyprobs --ppm --accmass --nonparam –expectscore”. ProteinProphet maintains default settings, “--maxppmdiff 2000000 --minprob 0.5”. FragPipe’s FDR filtering criteria are established as “--sequential --prot 0.01”.

Table S4. Parameter settings of Sage on different datasets.

Parameter Name	TTOF5600	TTOF6600	Orbitrap	timsTOF	PXD001468	PXD041271
precursor_tol (narrow search)	-50~50	-50~50	-10~10	-15~15	-10~10	-10~10
precursor_tol (open search)	-150 Da~ 150 Da	-150 Da~ 150 Da	-150 Da~ 150 Da	-150 Da~ 150 Da	-150 Da~ 150 Da	-150 Da~ 150 Da
fragment_tol	100	100	20	50	20	20
modifications	C: 57.02<br>M: 15.99	C: 57.02<br>M: 15.99	C: 57.02<br>M: 15.99	C: 57.02<br>M: 15.99	C: 57.02<br>M: 15.99	C: 57.02<br>M: 15.99<br>S: 79.97<br>T: 79.97<br>Y: 79.97
cleave_at/restrict	KR/P	KR/P	KR/P	KR/P	KR/P	KR/P

"precursor_tol" indicates the mass error of peptide parent ions, measured in ppm. In the open search mode, it is set to the default range of -150 Da to 500 Da.

"fragment_tol" represents the mass error of fragment ions, also measured in ppm.

"modifications" denotes the modification settings, with the format being "position: mass". When searching for phosphorylation modifications in the PXD041271 dataset, "STY" needs to be separately added as three individual positions.

"cleave_at/restric" indicates the theoretical enzyme cleavage settings for peptides, with cleavage sites set to "KR" and skipped sites set to "P". All other parameters are set to default values.

Materials and Methods (Text S1-S5)

Text S1. Optimal Binning Methods.

The inherent limitations in mass spectrometers result in a certain margin of error in the measured mass-to-charge ratio (m/z) data, conventionally expressed in parts per million (ppm). Assuming an instrumental error of t ppm and a fragment mass of m, the corresponding error is computed as $m \times t \times 10^{-6}$. However, the utilization of ppm for error calculation leads to varying error ranges for different m/z, complicating the generation of fragment inverted index table and significantly escalating computational complexity during the peptide-spectrum intersection calculations.

To expedite these intersection calculations, many peptide searching engines resort to data binning, a technique involving the transformation of decimal values into integers within an allowable error range. Under binning methodology, assuming a bin width of w, the transformed integer is represented as $[m \times w]$, where $[*]$ signifies a rounding function. By dividing this integer by the bin width, the decimal value is restored, resulting in an error of $m - [m \times w]/w$. The process of data binning facilitates the conversion of m/z into integers, thereby simplifying the intersection computation between peptides and experimental spectra by treating them as intersections between two sets of integers, notably reducing computational complexity.

However, the error generated by binning doesn't precisely match the instrument's error, and the bin width is typically a constant value. Consequently, binning algorithms may perform differently across different instruments. Here, we propose a straightforward and effective algorithm for determining the optimal bin width. By defining the user-set error as the target error, we calculate the mean square deviation between the actual error generated by various bin widths and the target error. The bin width with the smallest mean square deviation is selected as the final bin width for utilization. The optimization formula is expressed as:

$$w_{best} = \min_{w=[5,10,15,\ldots 100]} \sum_{i=1}^{N} \left(m_i - \frac{[m_i * w]}{w} - \frac{m_i \times t}{10^6} \right)^2$$

where, “w” represents the width of the bins, and ranges from 5 to 100, with intervals of 5. “N” signifies the total number of fragment ions contained within the experimental data. By searching and selecting the most suitable bin width, the goal is to minimize the error generated by the binning algorithm, making it as close as possible to the user specified ppm error.

Text S2. Optimization of Variable Modification Numbers.

The number of variable modifications determines the theoretical peptide count generated during the search process. Modifications are commonly divided into fixed and variable types. In calculating the mass of theoretical fragment-ions, fixed modifications indicate a definite change in the specified amino acid's mass, while variable modifications occur with a 50% probability. For instance, a peptide containing n variable modification sites could yield 2^n potential modified peptides. Variable modifications significantly increase the exponential growth of candidate peptides in searches. Most search engines limit variable modifications to a maximum of 3 per peptide, substantially restricting the peptide search space.

In order to maximize the peptide search space to discover new peptides and proteins, Dear-PSM considers all combinations of variable modifications. Specifically, assuming a peptide contains n variable modifications, Dear-PSM will calculate 2^n theoretical peptides generated by their combinations. As each additional variable modification increases the number of combinations exponentially, it significantly affects the computational complexity of peptide spectrum matching. Therefore, the number of variable modifications is the most crucial parameter in peptide spectrum matching.

While this parameter can be determined from the average theoretical peptide length and distribution of modification numbers, experimental optimization is still required to validate its impact on search results. Thus, a file from the PXD041271 dataset was selected as test data in this study. Narrow window searches were performed using Dear-PSM on the test data, and the impact of different modification numbers on search results was compared.

As shown in **Figure S11**, when variable modifications were not considered, Dear-PSM only identified 510 peptides. With an increase in the number of variable modifications, the number of peptides discovered by Dear-PSM also increased. When the numbers of variable modifications were 5 and 10, Dear-PSM identified 4653 and 6602

peptides, respectively, which were 9 times and 13 times higher than when there were no modifications. This indicates that the number of variable modifications significantly influences the search results for special modification data. When the number of variable modifications exceeded 15, the number of peptides identified by Dear-PSM stabilized. At this point, further increasing the modification number only significantly increased search time without significantly increasing the number of peptides. Therefore, after balancing search time and search space, Dear-PSM set the default value for the number of variable modifications to 20.

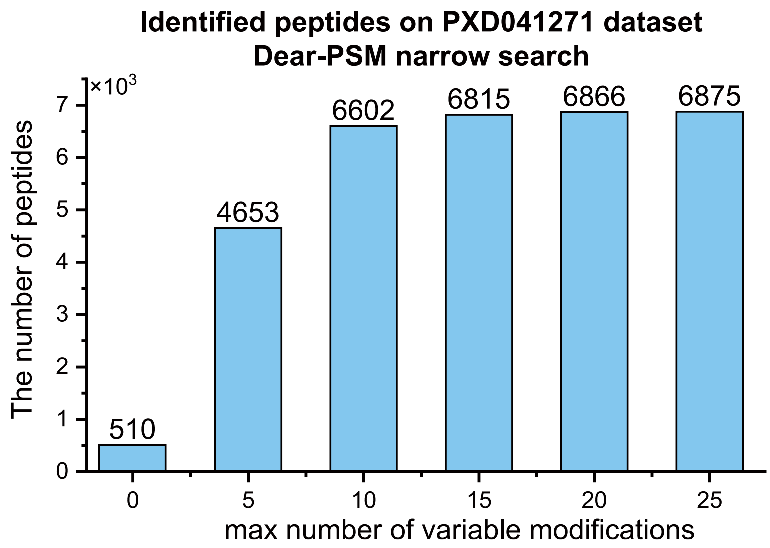

Figure S11. The Impact of Variable Modification Numbers on Search Results. Dear-PSM conducted searches on the phosphorylation-modified PXD041271 dataset using narrow window search mode. The horizontal axis represents the maximum number of variable modifications per peptide, while the vertical axis represents the number of identified peptides.

Text S3. False Discovery Rate Control.

False discovery rate (FDR) control is pivotal in proteomics. By default, Dear-PSM filters at a 1% protein-level FDR during peptide validation, and the users can opt for a 1% peptide-level FDR setting. In the standard target-decoy search strategy, FDR is computed as the ratio of decoys (d) above a threshold to targets (t):

$$\mathrm{FDR} = \frac{d}{t}$$

By adjusting FDR, corresponding Q-values are derived, maintaining them below 1% to manage FDR within that threshold. Peptide-level FDR is determined by sorting deep learning judgment scores in descending order. For protein-level FDR, the best peptides of a protein are utilized to compute protein scores, using the LPGM score[39] as the protein probability algorithm:

$$LPGM = -\log_{10}(1 - (1 - 10^{LPM})^{n}),$$

$$LPM = \max_{i=1,2,\ldots n}(-\log_{10}\frac{d+k}{D}),$$

where d represents the number of decoy peptides, and D indicates the total decoy peptides in the protein database. For decoy peptides, k = -0.5, while for target peptides, k = +0.5. And n denotes the number of matched peptides in the search results.

Text S4. Parameter Optimization of Retention Time Prediction Model.

Sage incorporates retention time information into PSM confidence estimation using a least squares regression model to more accurately determine misidentified peptides. Dear-PSM employs a deep learning model to predict peptide retention time, which serves as input for subsequent classification models to improve accuracy. To train the deep learning model, target peptides filtered at a 1% FDR threshold were selected, and training and validation sets were constructed. The trained model was then used to predict retention time for all search results.

To input peptide amino acid sequences into the deep learning model, character-based peptides are encoded into numerical vectors. Initially, a 20-dimensional vector is generated where each element represents the frequency of occurrence of each amino acid. Then, the first and last two amino acids of the peptide are encoded into one-hot vectors of length 20, with the occurring amino acid represented as 1 and the non-occurring amino acid as 0. Finally, these three vectors are concatenated into a single 60-dimensional vector, serving as input data for the neural network, thus completing the peptide encoding process.

We constructed a multi-branch deep neural network to predict peptide retention time. The network consists of four branches, each composed of Fully Connected (FC) layers. Specifically, the first branch contains one FC layer with a dimension of 128, the second branch contains two FC layers with dimensions of 192 and 256, the third branch contains two FC layers with dimensions of 32 and 64, and the last branch contains three FC layers with dimensions of 192, 64, and 16, respectively. ReLU is used as the activation function for each FC layer. A dropout operation is applied to each branch's output to prevent overfitting. The outputs of the four branch networks are concatenated into a 464-dimensional vector (128+256+64+16=464), which is then connected to a single-dimensional output layer (**Figure S1**). The expression for the mean squared error loss function is shown below:

$$MSE\ loss = \frac{1}{N}\sum_{i=1}^{N}(pred_i - t_i)^2$$

where $pred_i$ represents the predicted value, t_i is the true value, and N denotes the number of input vectors.

The training of deep learning models mainly involves several key factors: the number of neurons, the choice of optimizer, and the training batch size. The number of neurons, representing the parameters of the network, affects the model's ability to extract features and its generalization ability. More neurons lead to stronger expressive power but may also cause overfitting. The optimizer used to optimize the objective function is a critical component of deep learning, with different optimizers exhibiting different convergence speeds and stability. The batch size affects the model's generalization ability, with larger batch sizes typically leading to better generalization.

To determine the specific values of these key parameters, this paper selects the LFQ_Orbitrap_DDA_QC_01.raw file from the PXD028735 dataset as the experimental test data. Using a dataset of 43789 target peptides filtered at 1% FDR, 90% of the data is randomly chosen as the training set, while the remaining 10% serves as the validation set.

Based on the network branch structure shown in **Figure S12**, this paper designs four models with different numbers of neurons. These models consist of single-layer, two-layer, two-layer, and three-layer fully connected layers from left to right. The number of neurons in the fully connected layers is the parameter to be optimized.

Since predicting peptide retention time is a regression problem, we evaluate the performance of models with different parameter configurations using three mainstream regression model metrics. The evaluation metrics include the coefficient of determination (R-Square, R^2), mean absolute error (MAE), and root mean square error (RMSE). The calculation formulas for these three metrics are as follows:

$$R^2 = 1 - \frac{\sum_{i=1}^{N}(y_i - \hat{y}_i)^2}{\sum_{i=1}^{N}(y_i - \bar{y}_i)^2}$$

$$\mathrm{MAE} = \frac{1}{N}\sum_{i=1}^{N}|y_i - \hat{y}_i|$$

$$\mathrm{RMSE} = \sqrt{\frac{1}{N}\sum_{i=1}^{N}\|y_i - \hat{y}_i\|^2}$$

Where, y_i represents the experimental value, $\bar{y}_i$ represents the mean of the experimental values, $\hat{y}_i$ represents the predicted value, and N represents the number of samples.

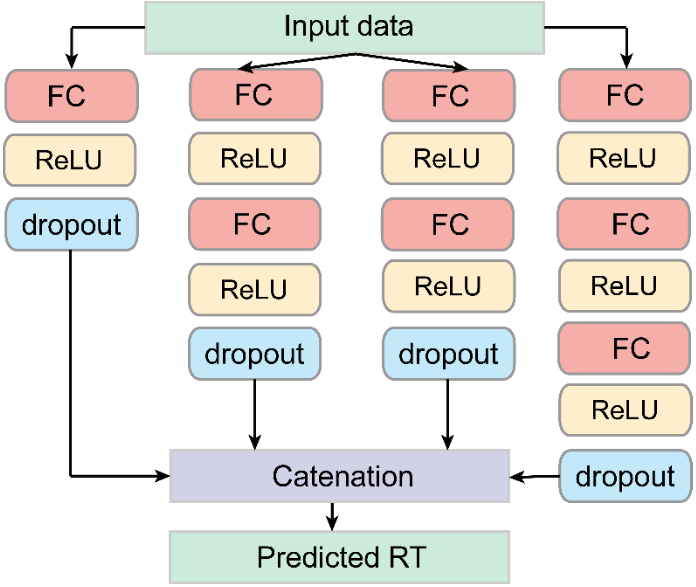

Figure S12. Diagram illustrating the structure of the multi-branch neural network. The red boxes represent fully connected layers where the number of neurons needs to be specified. Yellow boxes represent ReLU activation functions, while blue boxes represent dropout operations.

As shown in the table below, we set the number of neurons in the fully connected layers of the four branches from left to right. For example, in Model 1, the first branch contains a fully connected layer with 8 neurons; the second branch contains fully connected layers with 12 and 16 neurons; the third branch has fully connected layers with dimensions 2 and 4; and the fourth branch has fully connected layers with dimensions 12,

4, and 1, respectively.

Model Name	The number of neurons in the fully connected layers for each branch	R^2	MAE	RMSE
Model 1	8, (12, 16), (2, 4), (12, 4, 1)	0.8512	404.1977	612.9025
Model 2	32, (48, 64), (8, 16), (48, 16, 4)	0.8549	396.4485	605.1634
Model 3	128, (192, 256), (32, 64), (192, 64, 16)	0.8567	396.5115	601.2495
Model 4	256, (384, 512), (64, 128), (384, 128, 32)	0.8528	403.2962	609.4751

Based on the performance metrics presented in the table, Model 2 exhibits the optimal MAE, while Model 3 demonstrates superior R^2 and RMSE metrics. To further compare Model 2 and Model 3, we examined their loss function descent curves on the validation set. As depicted in **Figure S13**, the loss function of Model 3 on the validation set is lower than that of Model 2, indicating better training performance for Model 3. Therefore, we select Model 3 as the deep learning model for predicting retention time.

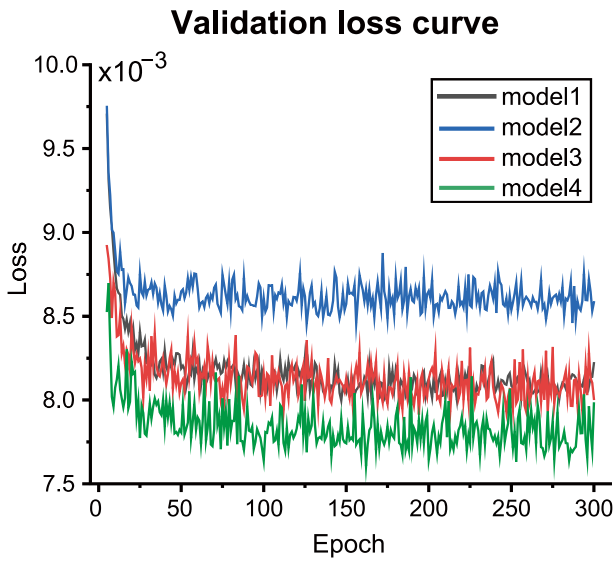

Figure S13. Validation set loss function curves for different models. The horizontal axis represents the number of training iterations, while the vertical axis represents the value of the loss function. The gray, blue, red, and green curves correspond to Model 1 through Model 4, respectively.

Next, we tested the impact of different batch sizes on the training and validation set loss functions. As shown in **Figure S14**, as the batch size increased from 64 to 1024, both the training and validation set loss functions continuously decreased. When the batch size reached 1024, there was a significant decrease in the validation set loss function. This indicates that increasing the batch size can lead to better generalization. Therefore, we set the default batch size to 1024 in this study.

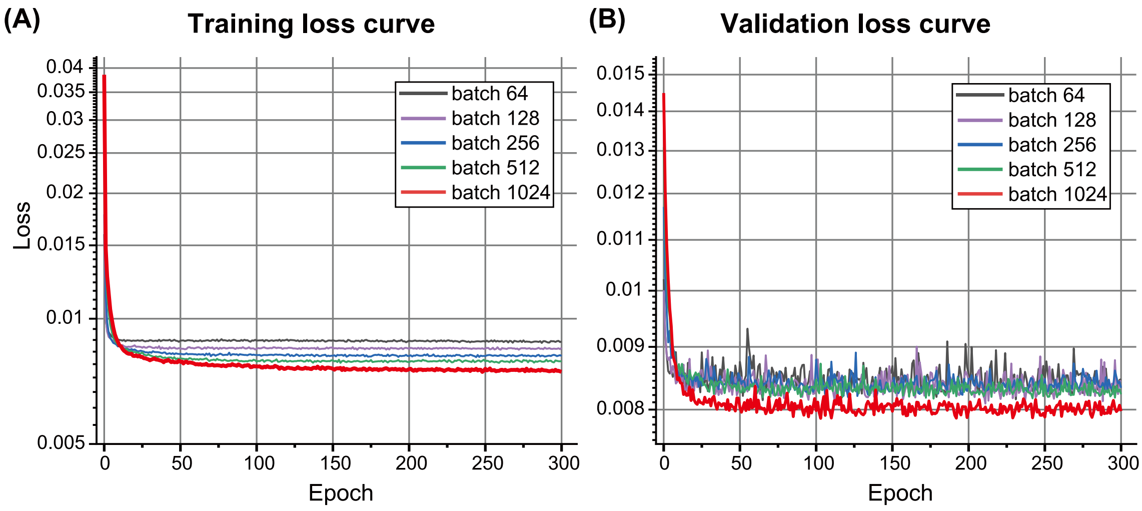

Figure S14. Training and validation set loss function curves for different batch sizes. The horizontal axis represents the number of training iterations, while the vertical axis represents the value of the loss function. Each curve of varying color represents a different batch size.

After fixing the model parameters, we compared the performance of six mainstream deep learning optimizers on the training and validation sets. The optimizers were tested using their default parameters. The optimizers included Stochastic Gradient Descent (SGD), Averaged Stochastic Gradient Descent (ASGD), Adaptive Moment Estimation (Adam), Weight Decay Regularization in Adam (AdamW), Adaptive Delta Algorithm (Adadelta), and Root Mean Square Propagation (RMSProp).

As illustrated in **Figure S15**, Adam exhibited the fastest convergence of the loss function on the training set, while AdamW achieved the minimum loss function value. On the validation set, Adam demonstrated the smallest loss function value and rapidly converged to a stable value, whereas AdamW showed an increasing trend in the loss function value on the validation set, indicating overfitting.

Therefore, we employed the Adam optimizer to optimize the mean squared error loss function, with a learning rate set to 0.001 and parameters β_1 and β_2 set to 0.9 and 0.999, respectively.

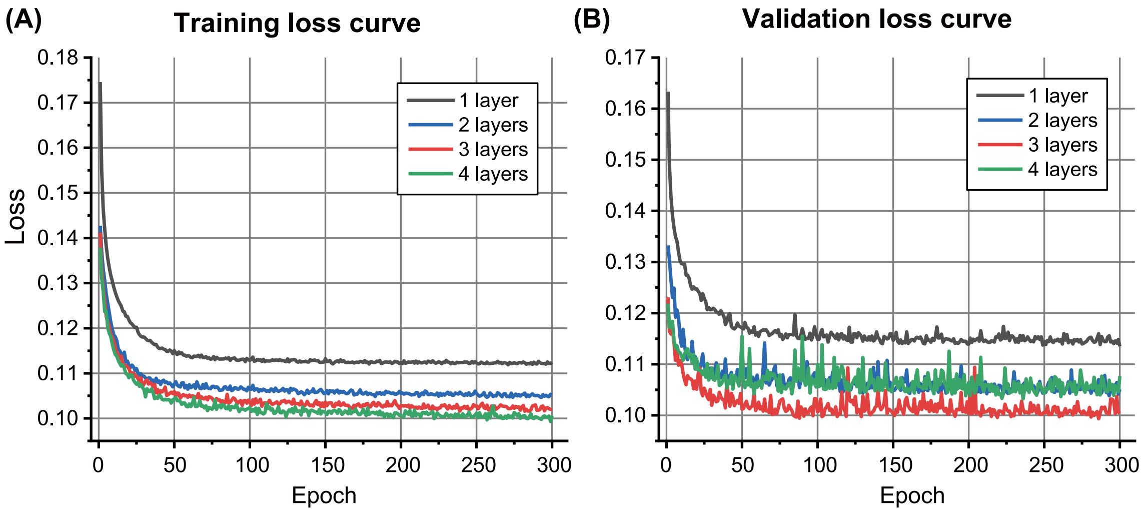

Figure S15. Loss function curves of different optimizers on the training and validation sets. The horizontal axis represents the number of training iterations, while the vertical axis denotes the value of the loss function. Each curve of a different color represents a distinct optimizer.

Text S5. Parameter Optimization of the Discriminant Score Model.

Deep learning has demonstrated remarkable performance in classification tasks, prompting its application in peptide validation. Within our framework, peptide validation is construed as a binary classification problem. Identified target peptides are designated as positive samples, denoted with a label of 1, while decoy peptides serve as negative samples, labeled as 0. To train the deep learning model, we first filter target peptides meeting a 1% PSM-level FDR threshold as positive samples, alongside using all decoy peptides as negative samples, constructing both training and validation datasets. Subsequently, this trained deep learning model is employed to evaluate all peptides, producing scores for each PSM. In scenarios where the count of target peptides falls below 1000 within smaller datasets, the linear discriminant analysis (LDA) model from machine learning is employed for binary classification of all peptides, producing scores for each PSM.

Enhancing the model's classification aptitude, diverse data attributes derived from search outcomes serve as feature vectors for model input. These encompass hyper-score, xcorr-score, retention time, average mass deviation of matched fragments, peptide mass, and other pertinent features. Detailed descriptions of these input features are provided in the.

We constructed a fully connected deep neural network. The architecture consists of three hidden layers, each housing 512 neurons and employing Rectified Linear Unit (ReLU) nonlinear activation functions. Dropout layers integrated after each hidden layer serve to mitigate overfitting. The output layer encompasses a solitary neuron using a Sigmoid function as the transfer function (**Figure 2B**). During the computation of individual PSM scores, the Sigmoid function is omitted from the output neuron to prevent score saturation at the extremes. We applied the Adam optimizer to optimize the Binary Sigmoid Cross Entropy loss function, setting the learning rate to 0.001 and parameters β_1 and β_2 to 0.9 and 0.99, respectively. The expression for the loss function is as follows:

$$Loss = -\frac{1}{N}\sum_{i=1}^{N} t_i \log f(x_i) + (1 - t_i)\log[1 - f(x_i)],$$

where x_i represents the input feature vector, t_i is the label of x_i. And N indicates the number of input vectors, $f(*)$ is the deep neural network.

Optimization of the multi-layer fully connected network involves tuning the network layers, the number of neurons in each layer, and selecting an appropriate optimizer. Similar to the parameter optimization process for predicting retention time, the PXD028735 dataset is utilized as experimental test data. All decoy peptides from the Dear-PSM output are considered as negative samples, while peptides meeting the 1% FDR threshold filter are regarded as positive samples. The dataset is split into training and validation sets in a 9:1 ratio.

Initially, the performance of four models with varying numbers of hidden layers is tested on both the training and validation sets. As depicted in **Figure S16**, the loss functions of all four models decrease and converge after training, indicating successful model training without overfitting. Although the four-layer network exhibits slightly lower loss on the training set, its performance on the validation set is inferior to that of the three-layer network. Therefore, we opt to utilize a three-layer fully connected network as the deep learning model for computing discriminant scores.

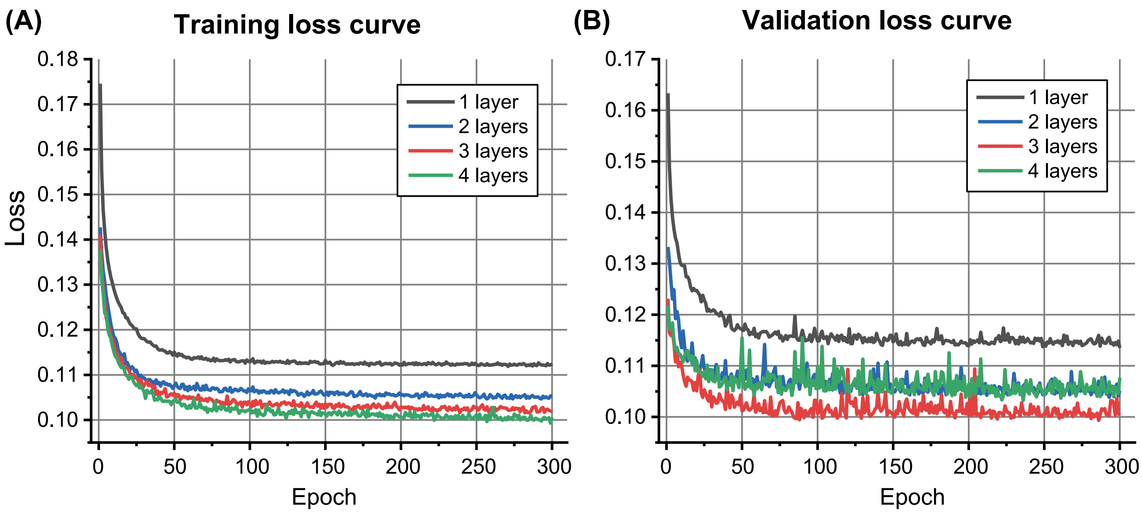

Figure S16. Loss function curves of models with different numbers of layers. The x-axis represents the number of training iterations, while the y-axis represents the loss function value. The curves in gray, blue, red, and green respectively represent neural networks containing 1 to 4 layers of fully connected layers.

Then, we further tested the influence of the number of neurons on the model performance. Since computing the discriminant score is considered a binary classification problem, this study employs five mainstream binary classification model metrics to evaluate the performance of models with different parameter sizes. The evaluation metrics include Area Under the Curve (AUC), Accuracy, Recall, Precision, and F1 Score. Among these, AUC represents the area under the Receiver Operating Characteristic (ROC) curve, with a larger AUC value indicating better classifier performance. The calculation of these metrics is as follows:

$$\text{Accuracy} = \frac{TP + TN}{TP + TN + FP + FN}$$

$$\text{Recall} = \frac{TP}{TP + FN}$$

$$\text{Precision} = \frac{TP}{TP + FP}$$

$$\text{F1 Score} = \frac{2 \times \text{Precision} \times \text{Recall}}{\text{Precision} + \text{Recall}}$$

where TP represents True Positives, TN represents True Negatives, FP represents False Positives, and FN represents False Negatives.

As shown in the table below, although increasing the number of neurons does not lead to a significant improvement in performance, the model with 512 neurons per layer performs best on the validation set, achieving an AUC of 0.985 and an F1 score of 0.976. Therefore, this paper sets the number of neurons per layer in the multi-layer fully connected network to 512.

Model Name	The number of neurons per layer	AUC	Accuracy	Recall	Precision	F1 Score
Model 1	32	0.983	0.957	0.986	0.963	0.975
Model 2	128	0.984	0.955	0.985	0.962	0.973
Model 3	256	0.982	0.950	0.985	0.957	0.971
Model 4	512	0.985	0.958	0.996	0.956	0.976

In addition, we also compared the performance of different optimizers on the training and validation sets to select the most suitable one. Default parameters were used for all tests. As illustrated in **Figure S17**, the performance of different optimizers on the training set is similar, all achieving rapid convergence. The Adam optimizer attained the lowest loss function value on the validation set. However, AdamW, AdaDelta, and RMSProp optimizers exhibited an increasing trend in the validation set's loss function, indicating overfitting. Consequently, we opted for the Adam optimizer to train the multi-layer fully connected deep learning model.

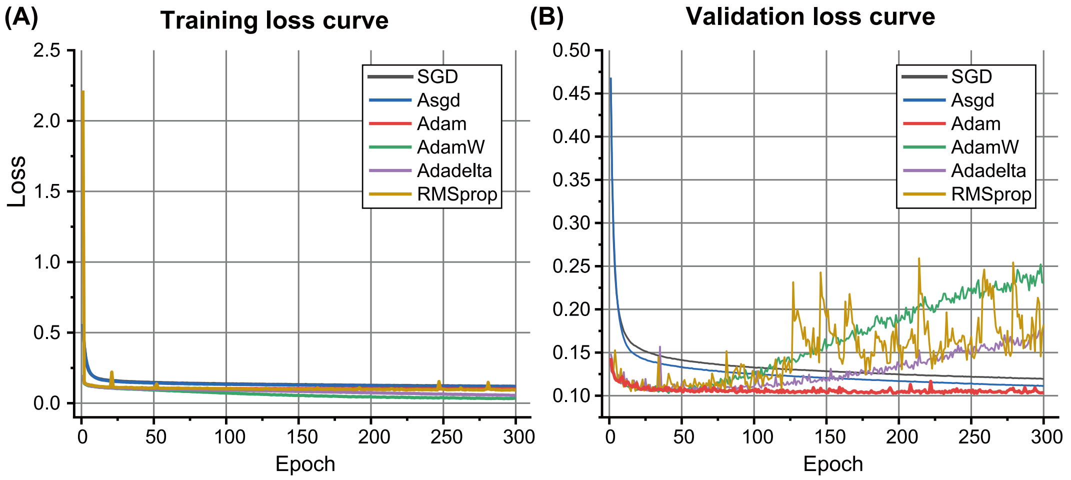

Figure S17. Loss function curves of models with different numbers of layers. The x-axis represents the number of training iterations, while the y-axis

represents the loss function value. Curves of different colors represent different optimizers.
